# Goblet Cell Hyperplasia Requires High Bicarbonate Transport To Support Mucin Release

**DOI:** 10.1038/srep36016

**Published:** 2016-10-27

**Authors:** Giulia Gorrieri, Paolo Scudieri, Emanuela Caci, Marco Schiavon, Valeria Tomati, Francesco Sirci, Francesco Napolitano, Diego Carrella, Ambra Gianotti, Ilaria Musante, Maria Favia, Valeria Casavola, Lorenzo Guerra, Federico Rea, Roberto Ravazzolo, Diego Di Bernardo, Luis J. V. Galietta

**Affiliations:** 1 U.O.C. Genetica Medica, Istituto Giannina Gaslini, Genova, Italy; 2 Department of Thoracic Surgery, University of Padova, Italy; 3 Telethon Institute of Genetics and Medicine, Pozzuoli, Italy; 4 Department of Biosciences, Biotechnologies and Biopharmaceutics, University of Bari, Italy; 5 DINOGMI, University of Genova, Italy

## Abstract

Goblet cell hyperplasia, a feature of asthma and other respiratory diseases, is driven by the Th-2 cytokines IL-4 and IL-13. In human bronchial epithelial cells, we find that IL-4 induces the expression of many genes coding for ion channels and transporters, including TMEM16A, SLC26A4, SLC12A2, and ATP12A. At the functional level, we find that IL-4 enhances calcium- and cAMP-activated chloride/bicarbonate secretion, resulting in high bicarbonate concentration and alkaline pH in the fluid covering the apical surface of epithelia. Importantly, mucin release, elicited by purinergic stimulation, requires the presence of bicarbonate in the basolateral solution and is defective in cells derived from cystic fibrosis patients. In conclusion, our results suggest that Th-2 cytokines induce a profound change in expression and function in multiple ion channels and transporters that results in enhanced bicarbonate transport ability. This change is required as an important mechanism to favor release and clearance of mucus.

The airway epithelium is covered by a periciliary liquid (PCL) whose thickness and composition are finely controlled by ion transport mechanisms^1^. PCL properties are important in the maintenance of innate defense mechanisms that protect the airway epithelium from pathogens and other noxious agents delivered to the airways with inhaled air^1^. In such processes, anion secretion plays a particularly important role. Chloride exit through channels at the apical membrane generates the driving force for sodium transport via the paracellular pathway. Net transepithelial transport of sodium chloride is then followed osmotically by water. This mechanism keeps the airway surface with the proper hydration required for mucociliary transport. In cystic fibrosis (CF), loss of function of CFTR^2^, a cAMP-regulated channel with a main role in epithelial anion secretion, leads to a shallow PCL^3^,^4^. Consequently, cilia, whose beating is required to move the mucus laying over the PCL, are immobilized. Accumulation of immobile mucus favors the survival and proliferation of bacteria^4^. Recently, bicarbonate has emerged as another important anion in addition to chloride. Bicarbonate secretion is required for the bactericidal activity of PCL^5^ and for the release and expansion of mucins^6^,^7^,^8^. Therefore, loss of CFTR-dependent bicarbonate transport is another factor that contributes to the genesis of CF lung disease.

Mucus accumulation in the airways is a feature of other respiratory diseases besides CF. In bronchial asthma, mucus hypersecretion and goblet cell hyperplasia, i.e. increase in the number of mucus-producing cells, are driven by the Th-2 cytokines IL-13 and IL-4^9^,^10^,^11^. Interestingly, these cytokines are also modulators of ion transport in bronchial epithelia. In particular, treatment *in vitro* of the epithelium with IL-4 or IL-13 for 24 hours upregulates chloride secretion and downregulates sodium absorption^12^,^13^. Such changes could be a required response through which the airway epithelium adapts to the increased abundance of mucus. In a previous study^14^, we found that treatment of epithelial cells with IL-4 increases the percentage of MUC5AC-positive goblet cells from 3% to 7% and 28% at 24 and 72 hours, respectively. The high percentage at 72 hours is reminiscent of the goblet cell hyperplasia occurring in human diseases characterized by mucus hypersecretion^9^,^10^,^11^. The aim of our study was to elucidate the modifications of ion transport mechanisms caused by IL-4 at 72 hours and their relationship with goblet cell hyperplasia. Using global gene expression profiling, short circuit current recordings, intracellular pH measurements, and protein immunodetection, we investigated the effects of IL-4 on ion transport at the functional and molecular levels. The results reveal a profound change in expression and function in multiple ion channels and transporters that results in enhanced bicarbonate transport ability. Importantly, CFTR appears to play a key role in this process since its loss of function impairs the mechanism of mucin release.

## Results

### Modulation of ion transport by IL-4

**Figure 1:**
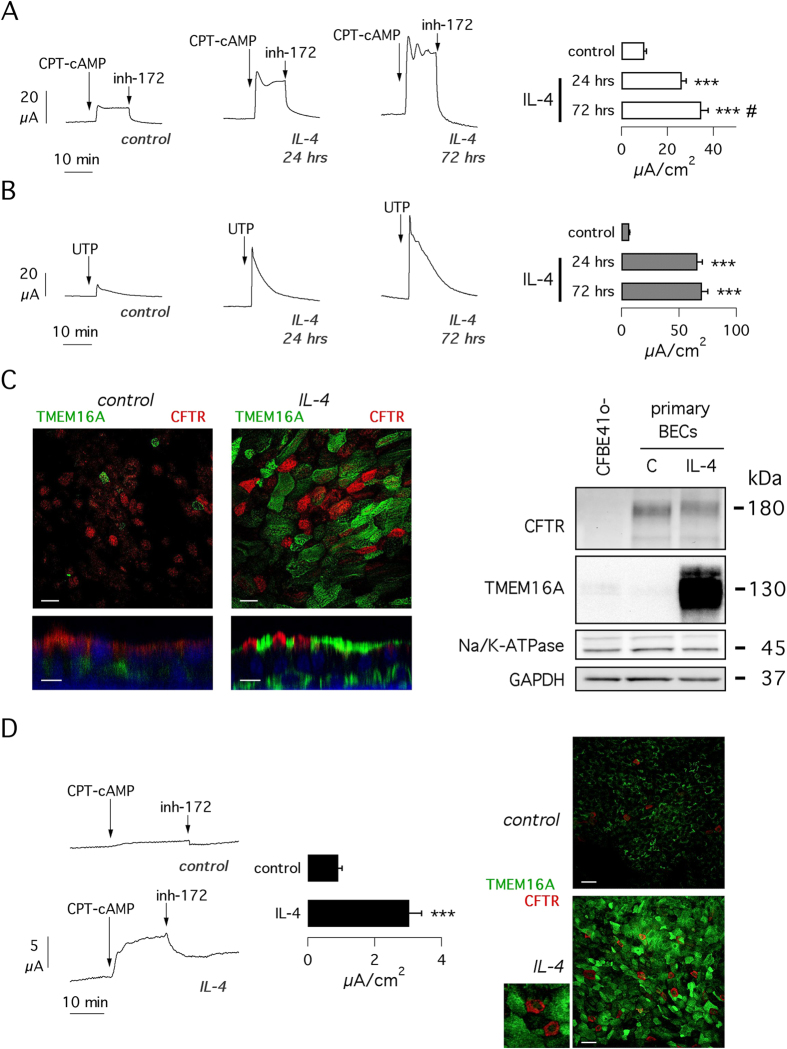
Upregulation of CFTR and TMEM16A function by IL-4. (**A,B**) Representative traces (left) and bar graphs (right) showing CFTR- and TMEM16A/CaCC-dependent currents measured by short-circuit current technique in human bronchial epithelial cells kept under control conditions or incubated for 24 or 72 hrs with IL-4 (10 ng/ml). CFTR currents were first activated with CPT-cAMP (100 μM) and then blocked with CFTR_inh_-172 (10 μM). TMEM16A/CaCC currents were instead activated with 100 μM UTP (in the presence of the CFTR inhibitor). Bar graphs report the size of the current drop induced by CFTR_inh_-172 (**A**) or the maximal amplitude of the current elicited by UTP (**B**). Data are the mean ± sem of 13–21 experiments (BE37 and BE63 cells). ***p < 0.001 vs. control. ^#^p < 0.05 vs. IL-4 for 24 hrs. (**C**) Detection of TMEM16A and CFTR proteins by immunofluorescence (left) and by western blot (right). Cells were treated with and without IL-4 or 72 hrs. Immunofluorescence images show *xy* (top; scale bar 20 μm) or *xz* (bottom; scale bar 10 μm) sections. In western blots, Na^+^/K^+^-ATPase β1 and GAPDH were also revealed as controls. As expected, the C464.8 antibody against Na^+^/K^+^-ATPase β1 revealed two bands. Western blot results for CFTR and TMEM16A are presented as cropped images. Full-length images are shown in Supplementary Fig. 1. (**D**) Short-circuit recordings (left) and immunofluorescence (right) from bronchial epithelial cells obtained from F508del/F508del CF patients. CFTR currents are significantly increased by IL-4 treatment (n = 15–16; p < 0.001; BE43, BE49, and BE91 cells). TMEM16A and CFTR expression, detected by immunofluorescence (scale bar 30 μm), is increased by IL-4 also in CF cells. The small image is an enlargement of the cells treated with IL-4 to show in more detail CFTR localization.

For our study, we used bronchial epithelial cells from two individuals, BE37 and BE63, which required lung transplantation due to pulmonary hypertension and idiopathic pulmonary fibrosis, respectively. We chose these cells as the closest to those of healthy controls. Indeed, the two diseases affect the distal part of the lungs and do not damage the epithelium of the main bronchi. We measured transepithelial ion transport properties in cells treated with IL-4 (10 ng/ml) for 24 and 72 hours. Figure 1 shows data obtained from short-circuit current experiments on well differentiated bronchial epithelia (cells plated on porous membrane and kept under air-liquid condition for three weeks). After blocking Na^+^absorption with amiloride (not shown), cells were stimulated with CPT-cAMP to induce phosphorylation and hence activation of CFTR (Fig. 1A). The resulting current was strongly sensitive to CFTR_inh_-172, a potent and selective CFTR inhibitor. In the presence of this inhibitor, apical application of UTP generated a very fast current increase that reached a maximum in a few seconds and then declined to pre-stimulation levels in 10–20 minutes (Fig. 1B). The effect of UTP is mediated by intracellular Ca^2+^ mobilization that leads to transient activation of TMEM16A Cl^−^ channels^15^).

Treatment with IL-4 promoted a marked increase in cAMP- and Ca^2+^-activated Cl^−^ secretion, as indicated by the amplitude of the currents blocked by CFTR_inh_-172 (Fig. 1A) or activated by UTP (Fig. 1B), respectively. CFTR-dependent current was increased 2.6-fold at 24 hours and 3.5-fold at 72 hours, with the two values being significantly different (Fig. 1A). The increase in UTP-dependent current was instead essentially the same (nearly 10-fold) at the two times of IL-4 treatment (Fig. 1B). It is interesting to note that in cells receiving IL-4 for 72 hours, CFTR currents initially showed oscillations that progressively disappeared (Fig. 1A).

We looked at the level of expression of CFTR and TMEM16A proteins with immunofluorescence and western blot techniques (Fig. 1C). With confocal microscopy, we found that IL-4 treatment elicited a strong upregulation of TMEM16A. Immunofluorescence detection of CFTR also revealed an increase in signal although less dramatic than TMEM16A. Both proteins appeared to be localized at the apical side of cells but, importantly, never within the same cell (Fig. 1C, left). In agreement with immunofluorescence and functional data, western blot experiments revealed that TMEM16A protein was strongly upregulated by IL-4 (Fig. 1C, right, and Supplementary Fig. 1). In contrast, the extent of CFTR protein expression by western blot appeared to be unaltered by the cytokine. We only noted a slight change in mobility that could reflect a modification of the pattern of glycosylation (Fig. 1C, right). We quantified mRNA levels by real time RT-PCR. CFTR mRNA was not altered by IL-4, whereas TMEM16A mRNA was upregulated 7-fold.

To further support the conclusion that the cAMP-dependent current is due to CFTR, we used cell from CF patients. In particular, we used cells from patients homozygous for the F508del mutation, which causes a severe defect in CFTR protein trafficking and a more than 90% decrease in CFTR function. As expected, the cAMP-dependent current was markedly reduced in CF cells, including those treated with IL-4 (Fig. 1D). However, we noted that IL-4 treatment for 72 hours induced a nearly three-fold increase in CFTR function, an effect proportionally similar to that of IL-4 in non-CF cells. By immunofluorescence, we found that IL-4 increases CFTR protein expression also in CF cells (Fig. 1D). Close inspection of microscopic images reveals that a large fraction of CFTR signal is intracellular given the perinuclear pattern of staining (Fig. 1D, enlarged image).

### Upregulation of ion channels and transporters by IL-4

**Figure 2:**
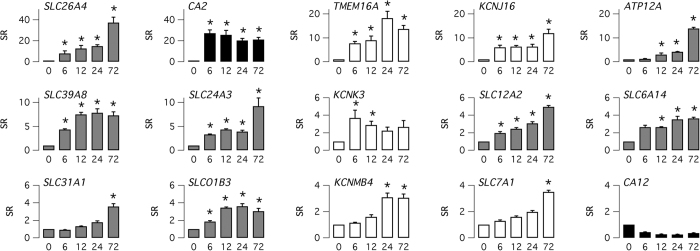
Upregulation of channels and transporters expression by IL-4. Bar graphs report the relative change in expression (y axis: signed ratio, SR) of the indicated genes after treatment of bronchial epithelial cells with IL-4 (10 ng/ml) for 6, 12, 24, and 72 hrs. Data were obtained by microarray analysis on three separate bronchial cell preparations (BE37 cells). The asterisks indicate a significant increase in expression relative to control cells (FDR < 0.05; data, including signed ratio and FDR, are also reported in Supplementary Tables 1–4 for the top 200 upregulated genes).

To determine the extent and time-course of gene expression changes elicited by IL-4, we extracted the RNA from cells treated at different time points, from 6 to 72 hours. Given the complexity of the study, involving four different time points and three separate cell preparations, we chose to use the cells from a single individual, BE 37. The RNA samples were analyzed by microarray hybridization (Fig. 2; GEO Access Number: GSE78914). We were particularly interested in the expression of genes involved in transepithelial ion transport. As shown previously by us, IL-4 markedly increases the expression of the TMEM16A chloride channel^15^ and of SLC26A4 (a.k.a. pendrin), an electroneutral anion exchanger^16^. The new data reveal that TMEM16A and pendrin upregulation is already detectable at 6 hours and that expression continues to increase although with a different time-course (Fig. 2). Pendrin has the strongest expression at 72 hours with a 37-fold increase (FDR < 10^−6^) compared to untreated cells. Instead, TMEM16A expression shows a peak at 24 hours (18-fold, FDR < 10^−4^) followed by a slight decrease at 72 hours. Importantly, microarray analysis revealed the upregulation of other ion transport systems. Some genes showed a relatively delayed response to IL-4 with a particular hyperexpression at 72 hours. This is the case of the genes coding for ATP12A, SLC31A1, KCNMB4, and SLC7A1 (Fig. 2). ATP12A is the non-gastric form of H^+^/K^+^-ATPase, responsible for H^+^ secretion at the apical membrane of epithelial cells^17^. SLC31A1 is a high-affinity copper transporter. KCNMB4 serves as a β subunit of the large conductance Ca^2+^-activated K^+^ channel. SLC7A1 is a high affinity cationic amino acid transporter. Other genes showed a more rapid induction by IL-4. This list includes genes coding for: KCNJ16, a pH-sensitive K^+^ channel^18^; SLC39A8, a bicarbonate-dependent zinc and iron transporter^19^; SLC24A3, a K^+^-dependent Na^+^/Ca^2+^ exchanger^20^; KCNK3, the TASK-1 two-pore K^+^ channel inhibited by extracellular acid^21^; SLC12A2, the NKCC1 co-transporter^22^; SLC6A14, the ATB^0,+^ amino acid transporter^23^; SLCO1B3, an organic anion transporter. Importantly, we also noted that IL-4 strongly induced the expression of the cytosolic carbonic anhydrase 2 (CA2), with a more than 20-fold (FDR < 10^−5^) upregulation already reached at 6 hours (Fig. 2). In contrast, another carbonic anhydrase, the membrane bound CA12, was downregulated. A more general list of top 200 genes upregulated by IL-4 is available in Supplementary Tables 1–4. It should be noted that, in agreement with real time RT-PCR data, CFTR expression was not affected by IL-4 treatment.

**Figure 3:**
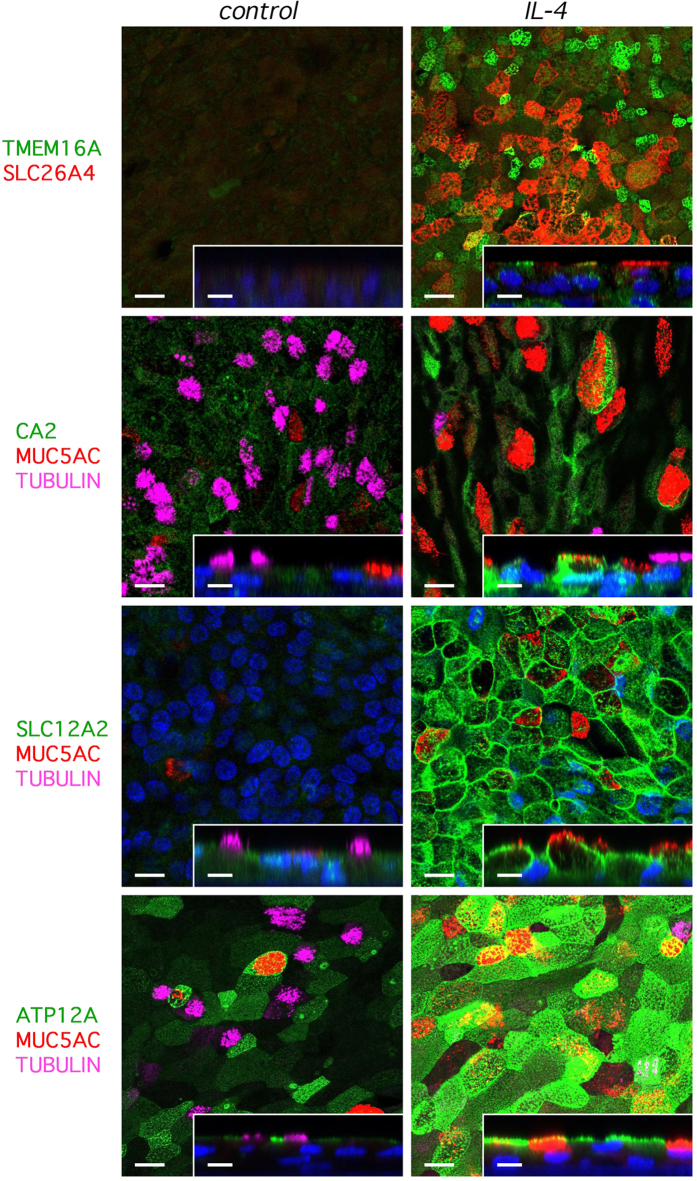
Immunofluorescence detection of proteins modulated by IL-4. Representative confocal microscope images show extent of expression and subcellular localization of TMEM16A, SLC26A4, carbonic anhydrase 2 (CA2), SLC12A2, and ATP12A (images taken from BE37 cells; similar results were obtained from BE63 cells). Whenever permitted by the combination of primary antibodies, acetylated tubulin and MUC5AC were also stained as markers of ciliated and goblet cells, respectively. Bronchial epithelia were kept under control conditions or treated with IL-4 for 72 hrs. Larger images: *xy* sections (scale bar: 20 μm). Inset: *xz* sections (scale bar: 10 μm). Images with a different scale of view are shown in Supplementary Fig. 2.

By immunofluorescence, we investigated the expression of selected proteins whose upregulation was suggested by microarray data. Whenever permitted by the compatibility of primary antibodies, we also stained the cells for acetylated tubulin or MUC5AC, markers of ciliated and goblet cells, respectively. Similarly to TMEM16A, SLC26A4 protein was markedly upregulated by IL-4, with localization in the apical membrane (Fig. 3). The two proteins showed co-localization in some cells but separate expression in many others. Immunodetection of CA2 revealed strong expression induced by IL-4, with a preferential localization in MUC5AC-positive cells (Fig. 3). Interestingly, SLC12A2 and ATP12A showed a more uniform distribution: they were strongly upregulated by IL-4 in most cells although with different subcellular localization (Fig. 3). As expected, SLC12A2 was in the basolateral membrane. ATP12A appeared in the apical region, with a more marked expression in non-ciliated cells. Expression of TMEM16A, SLC26A4, CA2, SLC12A2, and ATP12A is also shown in Supplementary Fig. 2 at a different scale of view. Upregulation of CA2 and SLC26A4 was also confirmed by western blot analysis (Supplementary Fig. 3).

### Bicarbonate transport in IL-4 treated epithelia

**Figure 4:**
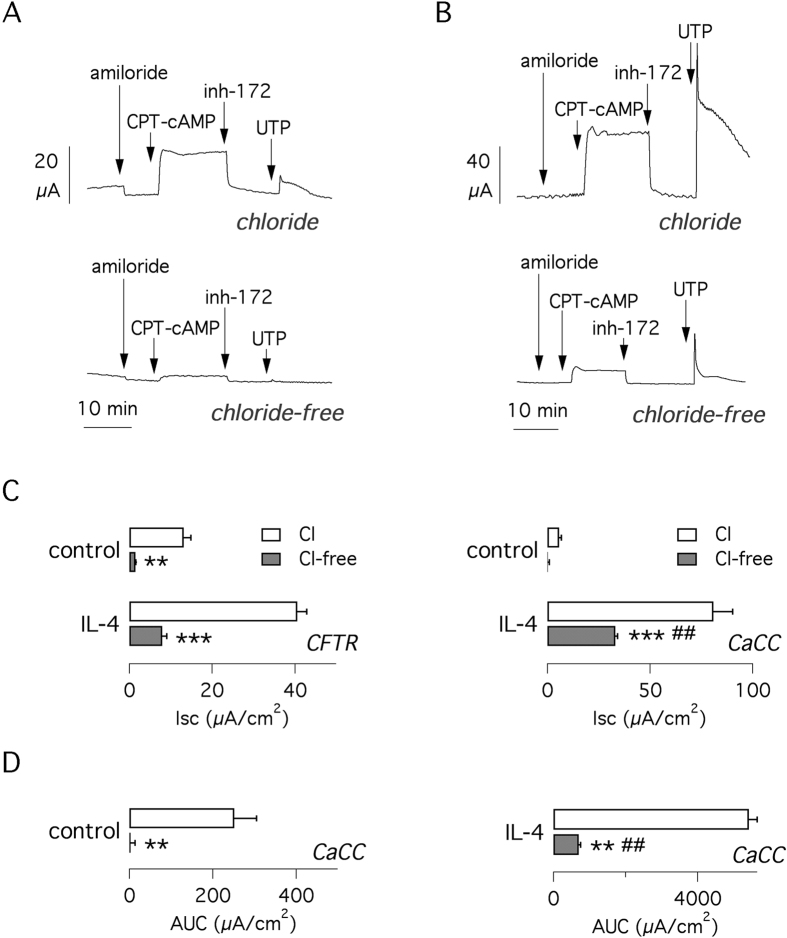
Effect of extracellular Cl^−^ removal. (**A,B**) Representative short-circuit current recordings showing CFTR and CaCC currents in cells kept under control conditions (**A**) or treated with IL-4 for 72 hrs (**B**). Note the different scale bars. Experiments were done in normal saline solution (top) or in the absence of Cl^−^ (bottom). (**C**) Bar graphs reporting CFTR (left) and TMEM16A data (right) under the different conditions. CFTR values are taken from the size of CFTR_inh_-172 effect. CaCC values represent the maximal current induced by UTP. (**D**) CaCC currents were also quantified as the area under the curve (AUC) of UTP-induced responses. **p < 0.01; ***p < 0.001 vs. currents with Cl^−^. ^##^p < 0.01 vs. cells not treated with IL-4 (n = 6 per condition; BE37 cells).

As indicated by microarray analysis, IL-4 stimulates the expression of several proteins involved in anion transport, possibly resulting in enhanced bicarbonate secretion. Therefore, we were particularly interested in the contribution of bicarbonate to cAMP- and Ca^2+^-activated currents. Accordingly, we carried out experiments in a Cl^−^-free solution, a condition that has been used to estimate net bicarbonate transport^24^. In untreated cells, the absence of extracellular Cl^−^ decreased the cAMP- and Ca^2+^-dependent currents to 11% and 5%, respectively (Fig. 4A,C). Treatment of cells for 72 hours with IL-4 changed the percentage of current remaining after Cl^−^ removal to 20% and 41%, respectively, representing a nearly two-fold and eight-fold proportional increase compared to untreated cells (Fig. 4B,C). Given the transient behavior of the Ca^2+^-dependent current, we also estimated its magnitude not as the size of peak but as area under the curve, AUC (Fig. 4D). Using this parameter, the UTP-activated Cl^−^-independent component appeared even larger in interleukin-treated compared to control cells (13% vs. 1% of the values measured in the presence of Cl^−^).

**Figure 5:**
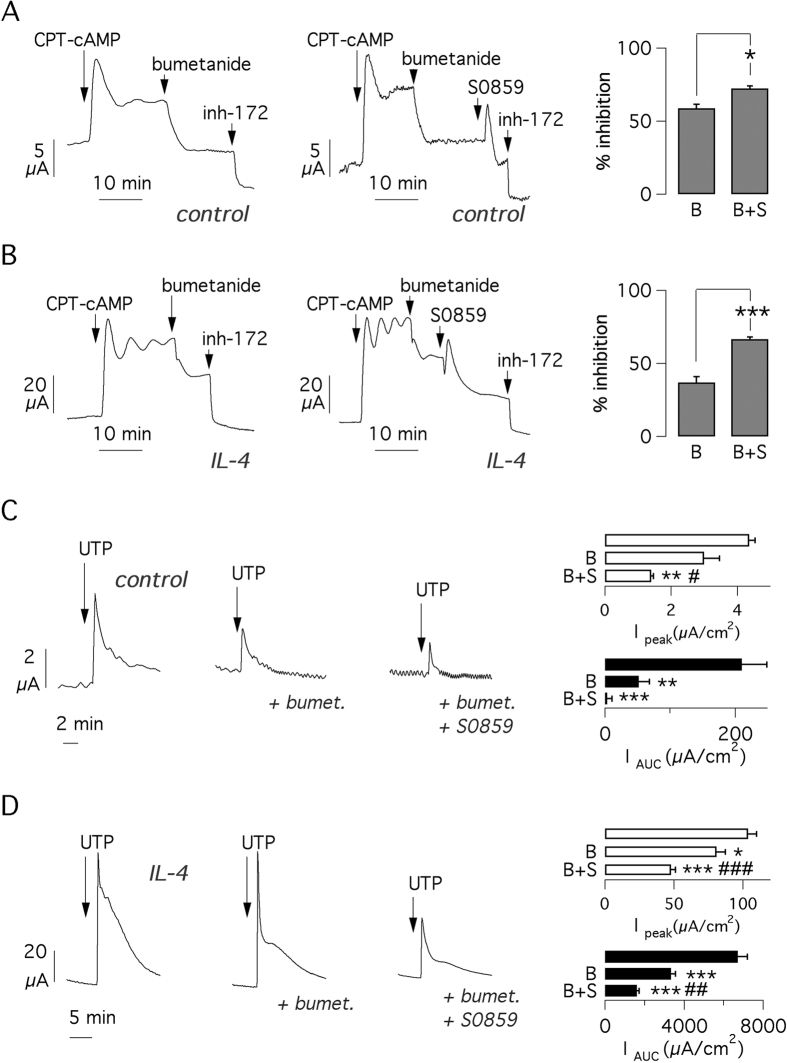
Effect of anion transport inhibitors. (**A,B**) Representative short-circuit current recordings and bar graphs reporting the effect of bumetanide and S0859 on CFTR-dependent currents in cells under control conditions (**A**) or treated with IL-4 for 72 hrs (**B**). The value of inhibition reported in bar graphs is calculated from the total CFTR current (current activated by CPT-cAMP minus the current remaining after CFTR_inh_-172). B: bumetanide. B + S: bumetanide + S0859. *p < 0.05; ***p < 0.001 vs. bumetanide alone (n = 8 per condition; BE37 cells). (**C,D**) Representative short-circuit current recordings and bar graphs reporting the effect of bumetanide and S0859 on UTP-induced currents in cells under control conditions (**C**) or treated with IL-4 for 72 hrs (**D**). Activity of TMEM16A/CaCC was quantified as current peak or as AUC. *p < 0.05; **p < 0.01; ***p < 0.001 vs. currents with no inhibitors. ^#^p < 0.05; ^##^p < 0.01; ^###^p < 0.001 vs. currents with bumetanide alone (n = 8 per condition).

To further investigate the changes elicited by IL-4, we used bumetanide as the blocker of the NKCC1 co-transporter and S0859 as a general inhibitor of basolateral bicarbonate transporters^25^. First, we studied the cAMP-activated current. As expected, given the role NKCC1 in supporting Cl^−^ secretion, bumetanide markedly decreased the cAMP-activated current in untreated cells (Fig. 5A). Subsequent addition of CFTR_inh_-172 allowed estimation of the residual CFTR-dependent component remaining after bumetanide. By considering the relative effects of bumetanide and CFTR_inh_-172, we calculated that NKCC1 inhibition removed ~60% of total secretion mediated by CFTR (Fig. 5A). In another set of experiments, we added S0859 after bumetanide. The representative trace in Fig. 5A shows that S0859 effect consisted of a small transient peak followed by a modest decrease of the current. The total inhibition obtained by S0859 plus bumetanide was only slightly higher than that elicited by bumetanide alone (Fig. 5A). In cells treated with IL-4 for 72 hours, the relative effects of bumetanide and S0859 were modified. The current inhibited by bumetanide was larger in absolute terms but the percent inhibition of total current was significantly smaller (<40%) if compared to that measured in untreated cells (Fig. 5A,B). This result suggested a higher contribution of other types of basolateral anion transporters. In agreement with this interpretation, S0859 appeared to induce a more marked inhibition in cells treated with IL-4 (Fig. 5B).

We also studied the effect of basolateral transporter inhibitors on the Ca^2+^-activated current. Apical UTP was given in the presence and absence of bumetanide alone or in combination with S0859. In untreated cells, the UTP-activated current, particularly if measured as AUC, was partially inhibited by bumetanide and almost totally blocked by bumetanide plus S0859 (Fig. 5C). A similar behavior (additive effect of bumetanide and S0859) was also seen in cells treated with IL-4. However, in treated cells the residual response to UTP in the presence of the two inhibitors was sensibly higher (Fig. 5D).

We noted that, even in the presence of bumetanide plus S0859, there were still residual cAMP- and Ca^2+^-dependent currents, particularly in cells treated with IL-4. To further inhibit transepithelial anion transport, we tested acetazolamide as an inhibitor of carbonic anhydrase. When given after bumetanide and S0859, acetazolamide further decreased the CFTR-dependent current (Supplementary Fig. 4). Total inhibition obtained with the three compounds together was ~85%. The UTP-activated current was also sensitive to acetazolamide. The peak induced by UTP in the presence of the triple combination of inhibitors was smaller than that measured with bumetanide plus S0859 (27.8 ± 2.2 vs. 47.9 ± 3.1 μA/cm^2^; Supplementary Fig. 4). As a control experiment, we first added CFTR_inh_-172 followed by CPT-cAMP. Importantly, after totally blocking CFTR with its selective inhibitor, no further decrease was seen with bumetanide, S0859, and acetazolamide (Supplementary Fig. 4). The only visible response remaining in the presence of CFTR_inh_-172 was the small transient current elicited by S0859. This response may be due to activation of Ca^2+^-activated Cl^−^ channels since it was inhibited by CaCC_inh_-A01 (Supplementary Fig. 4).

**Figure 6:**
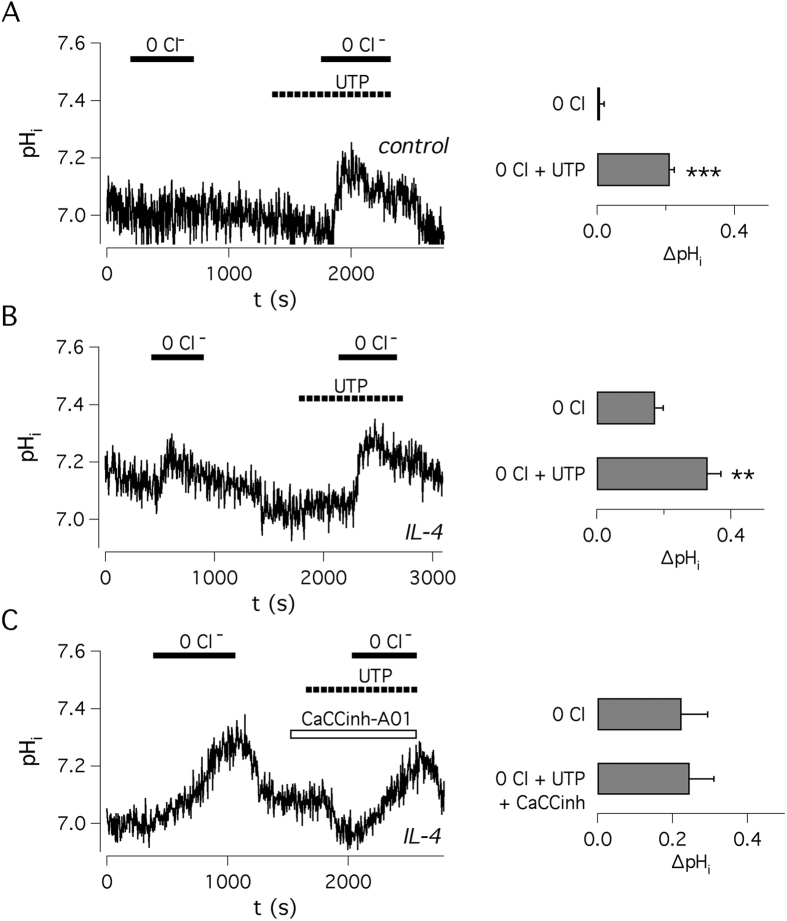
Intracellular pH measurements in bronchial epithelia. Representative traces show intracellular pH values measured with BCECF probe with and without apical Cl^−^. Bar graphs show changes in pH after removal of apical Cl^−^ in the absence and presence of apical UTP. (**A**) Bronchial epithelial cells without IL-4 treatment (n = 7). (**B**) Cells treated with IL-4 for 72 hrs (n = 7). (**C**) Cells treated with IL-4 and UTP applied in the presence of CaCC_inh_-A01 (n = 5). **p < 0.01; ***p < 0.001 (BE43 cells).

As indicated by our study, pendrin/SLC26A4 is one of the most upregulated genes by IL-4. However, pendrin activity cannot be detected in short-circuit current recordings since its mechanism of ion transport is electroneutral^16^. Therefore, we looked for evidence of bicarbonate transport by measuring intracellular pH with the BCECF fluorescent probe as done previously in Calu-3 cells^26^. To this aim, polarized cell monolayers loaded with BCECF were mounted in a specially-designed cuvette which allowed independent perfusion of the apical and basolateral sides. To assess the presence of a pendrin-mediated Cl^−^/HCO_3_^−^ exchange, we perfused the apical side with a Cl^−^-free solution. To remove the contribution of CFTR, which is also permeable to bicarbonate, we used CF cells and no cAMP stimulation. As shown in Fig. 1D, there is negligible contribution of CFTR under these conditions. In untreated cells under resting conditions, apical Cl^−^ removal caused no change in intracellular pH (Fig. 6A). In contrast, a significant alkalinization was observed in cells treated with IL-4 thus indicating the enhanced expression of an apical bicarbonate exchanger (Fig. 6B). Indeed, in the absence of extracellular Cl^−^ (replaced by gluconate) the exit of bicarbonate through an exchange mechanism is impeded. In the same experiments, we subsequently stimulated the cells with apical UTP. Under this condition, Cl^−^ replacement elicited an intracellular alkalinization that was larger in cells treated with IL-4 (Fig. 6A,B). This effect may result from entry of bicarbonate through Ca^2+^-activated Cl^−^ channels. To explain this effect, we have to consider that the electrogenic exit of Cl^−^ through the channels is increased after extracellular Cl^−^ removal. This causes a depolarization of membrane potential (inner side more positive) that causes enhanced bicarbonate entry. Importantly, in the presence of the inhibitor CaCC_inh_-A01, which blocks Ca^2+^-activated Cl^−^ channels, UTP-dependent alkalinization was not different from that elicited by Cl^−^ removal alone (Fig. 6C) suggesting that TMEM16A works synergistically with pendrin in secreting HCO_3_^−^.

**Figure 7:**
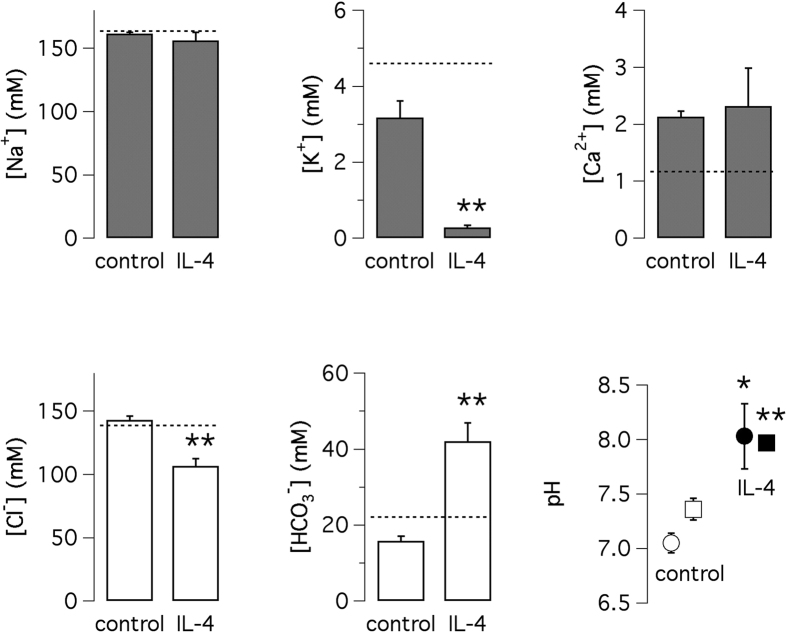
Alteration of apical fluid by IL-4. Ion composition and pH of apical fluid from cells kept under control conditions or treated with IL-4. A fixed volume (150 μl) of saline solution was applied to the apical side of epithelia and recovered after 48 hours. Dotted line shows the initial concentration of each ion in the original solution. For pH, values were determined with litmus strips (circles) or with pH-sensitive electrodes (squares). *p < 0.05; **p < 0.01 vs. control (n = 3–4; BE37 cells).

To more directly test the ability of bronchial epithelial cells to secrete bicarbonate, we carried out experiments in which the apical side of epithelia was covered with 150 μl of Krebs solution containing 128 mM Cl^−^ and 24 mM HCO_3_^−^. After 48 hours of incubation at 37 °C in a humidified 5% CO_2_ atmosphere, the apical fluid was collected to measure the ion composition (Fig. 7). We noted a remarkable difference between cells treated with and without IL-4. In untreated cells, Cl^−^ increased to 143 mM and HCO_3_^−^ decreased to 16 mM with respect to the initial concentration of each ion in the original solution. With IL-4, Cl^−^ concentration decreased to 107 mM whereas HCO_3_^−^ was accumulated to 42 mM (Fig. 7). In agreement with the high HCO_3_^−^ concentration, the apical fluid of cells treated with IL-4 was more alkaline as indicated by two different types of pH measurements (Fig. 7). Another significant change regarded K^+^: from the initial value of 4.6 mM, K^+^ concentration was moderately modified in control cells (3.2 mM) but markedly decreased (0.3 mM) in IL-4 treated cells (Fig. 7). In contrast, Na^+^ and Ca^2+^ concentration were not significantly altered. We were concerned that addition of liquid on the apical side for 48 hours could perturb the behavior of epithelia in terms of response to IL-4. Supplementary Fig. 5 shows that submerged cells still respond to IL-4 with a marked goblet cell hyperplasia. Control short-circuit current recordings, done after removal of apical fluid, also confirmed upregulation of CFTR and TMEM16A currents by IL-4.

**Figure 8:**
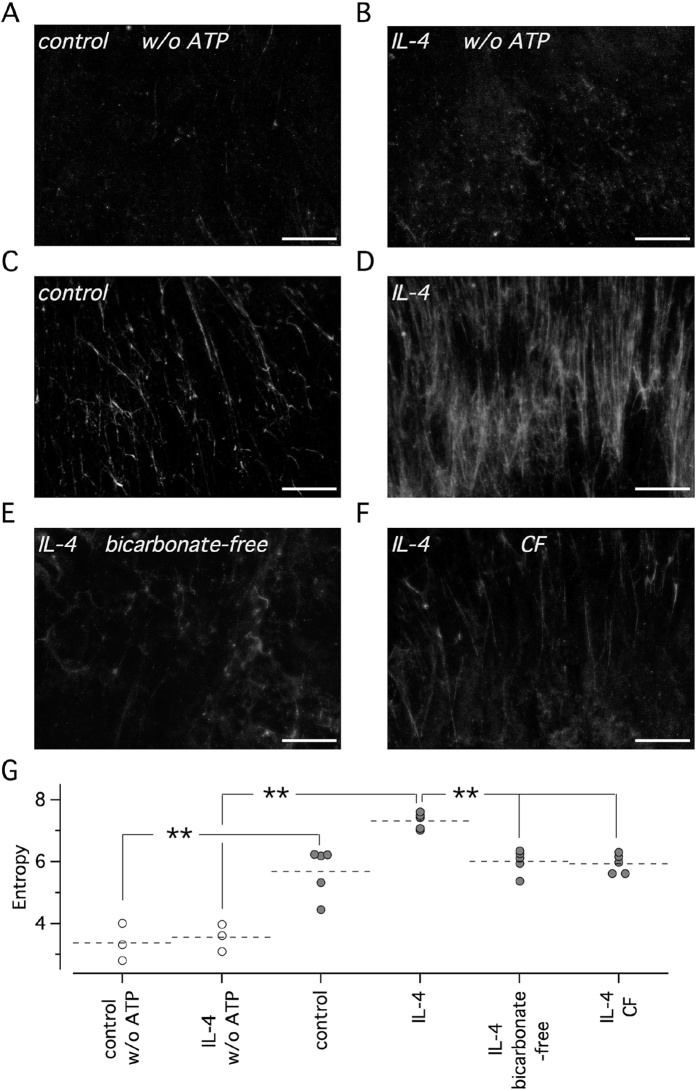
Mucus release by bronchial epithelia. (**A–F**) Representative images showing staining of mucus with fluorescent nanospheres added (50 μl of saline solution) on the apical side of tilted epithelia. Where indicated (**C–F**) the added solution contained ATP (100 μM) to stimulate mucus release. Experiments were done on epithelia treated with and without IL-4 (10 ng/ml) for 72 hours as indicated. Scale bar size is 500 μm. (**G**) Quantification of mucus strands for the different conditions (n = 3–6 per condition; BE37 and BE43 cells). The effect of IL-4 was statistically significant (p < 0.01).

We asked whether the upregulation of anion transport in cells treated with IL-4 plays a role in mucus release. For this purpose, we devised an assay in which ATP plus fluorescent nanospheres (in 50 μl saline solution) were added to the apical side of epithelia. Epithelia were kept tilted during the addition of fluid to allow unidirectional flow of solution by gravity. After removal of excess fluid, mucus stained by nanospheres was visualized by fluorescence microscopy. In control experiments, done in the absence of ATP, very little fluorescence was visible in cells either treated with and without IL-4 (Fig. 8A,B). This finding indicates that nanospheres do not bind to epithelial surface if mucus release is not stimulated. When ATP was included in the solution, epithelia not treated with IL-4 showed appearance of fluorescent filaments suggesting release of mucus (Fig. 8C). Filaments were strongly increased in number and intensity in cells treated with IL-4 for 72 hours (Fig. 8D). This pattern suggested formation of a complex network of mucus strands upon stimulation with ATP. Importantly, a marked reduction in mucus strands was detected when epithelia treated with IL-4 were previously exposed for three hours to a bicarbonate-free basolateral solution (Fig. 8E) or when experiments were carried out on CF epithelia treated with IL-4 (Fig. 8F). Summary of data is shown in Fig. 8G.

### Upregulation of SLC6A14

Although not directly connected to bicarbonate transport, we were intrigued by the upregulation of SLC6A14 by IL-4 (Fig. 2). SLC6A14 is responsible for Na^+^-dependent uptake of basic and neutral amino acids at the apical membrane^23^,^27^, and has recently been identified as a modifier of lung disease in CF^28^. We carried out short-circuit current experiments in which the apical side of epithelia was exposed to lysine or arginine (Supplementary Fig. 6). Amino acids elicited very small responses in untreated cells. In contrast, epithelia treated with IL-4 showed currents that rapidly appeared after amino acid addition.

## Discussion

Prolonged treatment of bronchial epithelial cells with IL-4 or IL-13 promotes marked morphological changes that recapitulate the goblet cell hyperplasia occurring in individuals affected by bronchial asthma^14^,^29^. Our study reveals that treatment with IL-4 results in a profound modification of ion transport mechanisms as indicated by analysis of gene expression with microarrays and by functional assays. At the transcriptome level, IL-4 causes the upregulation of several genes coding for ion channels and transporters. In addition to TMEM16A Cl^−^ channel and to pendrin/SLC26A4 anion exchanger, IL-4 increases the expression of SLC12A2 which codes for NKCC1. This basolateral co-transporter, whose upregulation was previously observed *in vivo* in asthmatic subjects^30^, generates intracellular accumulation of Cl^−^ by coupling its uptake with that of Na^+^ and K^+^. Therefore, NKCC1 is an important determinant of the driving force for Cl^−^ secretion. Importantly, as revealed by immunofluorescence, upregulation of NKCC1 by IL-4 occurs in ciliated as well as in goblet cells. Interestingly, other genes upregulated by IL-4 include the ATP12A proton pump, the pH-sensitive KCNJ16 and KCNK3 K^+^ channels, and the CA2 carbonic anhydrase, all proteins that may be involved in pH and bicarbonate homeostasis.

At the functional level, IL-4 strongly upregulates Ca^2+^- and cAMP-dependent Cl^−^ secretion. While the former process appears to be tightly correlated with overexpression of TMEM16A protein, the latter one appears surprising since CFTR protein expression was found to be unaltered in lysates of IL-4 treated cells. Furthermore, CFTR is specifically expressed in ciliated cells that actually undergo a significant reduction in epithelia treated with IL-4 for 72 hours^14^. To explain the upregulation of cAMP-dependent secretion, it can be hypothesized that the decrease in the number of CFTR-expressing cells is counteracted by upregulation of basolateral Cl^−^ transporters (e.g. NKCC1) that results in a higher driving force for Cl^−^. The higher driving force for Cl^−^ could also explain the increase in cAMP-dependent Cl^−^ secretion measured in CF cells. However, we cannot exclude that IL-4 also increases CFTR protein expression at the cell surface, a phenomenon that could depend on enhanced trafficking of the protein to the apical surface and/or reduced internalization.

Several lines of evidence suggest that a major effect of changes induced by IL-4 is the enhanced ability of epithelia to secrete bicarbonate. In particular, removal of extracellular Cl^−^ revealed residual transepithelial currents, representing bicarbonate secretion, that were upregulated by IL-4. Also, S0859 and acetazolamide, which affect intracellular bicarbonate concentration in different ways, caused additive inhibitory effects on cAMP- and Ca^2+^-dependent transepithelial currents. Furthermore, measurements of intracellular pH revealed an enhanced permeability to bicarbonate in IL-4 treated cells that may be due, at least in part, to pendrin expression in the apical membrane. Finally, measurements of apical fluid composition showed highly increased bicarbonate concentration, largely exceeding the one on the basolateral side, and, accordingly, a significant alkalinization. This finding remarks the increased capacity of epithelia treated with IL-4 to secrete and generate an asymmetrical distribution of bicarbonate.

Importantly, we found that bicarbonate transport in IL-4 treated cells has an important role in the mechanism of mucus release. When stimulated with a purinergic agonist, cells rapidly released mucus as indicated by staining with fluorescent nanospheres. This mechanism was significantly inhibited by keeping the cells in a bicarbonate-free basolateral solution. Furthermore, mucus release was similarly inhibited when the assay was carried out on CF cells. This result indicates that CFTR activity is particularly important under goblet cell hyperplasia conditions. Interestingly, a defect in mucus release was also recently shown in intestinal goblet cells of CF mice^31^. Such findings seem counterintuitive since CF disease is actually characterized by extracellular accumulation of mucus. However, it is possible that a CFTR-dependent primary defect impairs normal release of mucus that is later expelled in a more condensed state. In this respect, it is intriguing that CFTR has such an important role in rapid mucus release despite being selectively expressed in ciliated and not in goblet cells. It can be hypothesized that CFTR affects mucus by modulating the ion composition of the extracellular milieu. Alternatively, it is possible that a low but physiologically significant expression of CFTR in mucin granules of goblet cells^32^ is important for mucus release.

**Figure 9:**
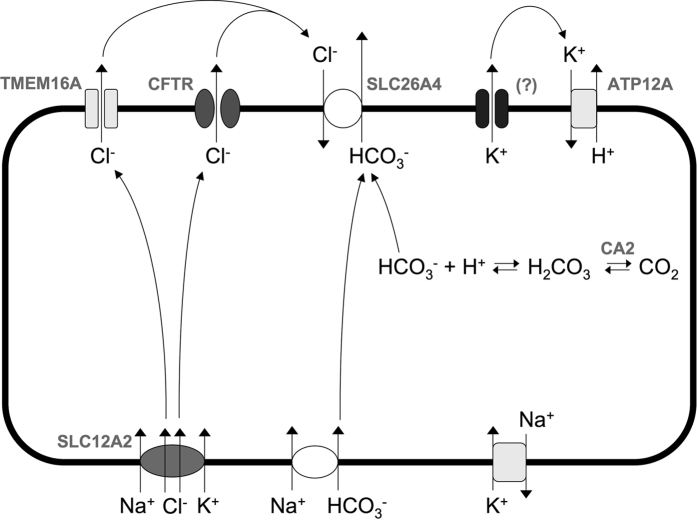
Mechanisms of anion secretion in bronchial epithelial cells exposed to IL-4. For simplicity, the cartoon shows all channels and transporters within the same cell although some components (e.g. CFTR and TMEM16A) are localized in separate cell types. The NKCC1 transporter (SLC12A2) promotes the intracellular accumulation of Cl^−^ that is then secreted through TMEM16A and CFTR Cl^−^ channels. Bicarbonate is accumulated inside the cell by means of basolateral transporters and by conversion from CO_2_. Pendrin then mediates the exchange of extracellular Cl^−^ with intracellular HCO_3_^−^. The apical membrane also contains the ATP12A K^+^/H^+^ pump and possibly a K^+^ channel. Secretion of K^+^ could be the mechanism controlling the acidification of apical fluid by ATP12A.

Summarizing, our results reveal profound changes in ion transport mechanisms occurring in bronchial epithelia under conditions that mimic goblet cell hyperplasia. Such changes appear to favor bicarbonate secretion and mucus release. Figure 9 shows a tentative model to depict the process of Cl^−^ and bicarbonate transport. Part of the information shown in the figure is supported by findings of this and previous studies. Other information, particularly the site of expression of some of the proteins induced by IL-4 (i.e. apical vs. basolateral membrane, ciliated vs. non-ciliated/goblet cells), remains to be resolved in future studies. Upregulation of NKCC1, essentially in all cells, indicates a general increase in the basolateral uptake of Cl^−^ that promotes its secretion through apical channels (CFTR and TMEM16A). Cl^−^ may be then recycled back through pendrin in exchange for bicarbonate. In this respect, it has previously been shown in Calu-3 cells that pendrin forms together with CFTR a functional unit that promotes bicarbonate secretion^26^. Regarding the source of intracellular bicarbonate, serving as a substrate for pendrin, our results suggest the contribution of S0859-sensitive basolateral transporters and CA2-mediated conversion from CO_2_. In the context of the model represented in Fig. 9, resulting in net bicarbonate secretion, the upregulation of ATP12A is particularly intriguing. ATP12A, a non-gastric form of H^+^/K^+^-ATPase, is expressed in the apical membrane of airway epithelia where it is involved in acidification of apical fluid coupled to K^+^ reabsorption^33^,^34^. The enhanced expression of ATP12A that we found in cells treated with IL-4 appears to be in contrast with the high bicarbonate levels and the alkaline pH measured in the apical fluid. However, we also found a low apical K^+^ concentration that could limit the activity of ATP12A thus allowing accumulation of HCO_3_^−^. Therefore, we can hypothesize that K^+^ exit at the apical membrane is the rate-limiting step controlling H^+^ secretion. The channel responsible for K^+^ secretion remains to be identified although possible candidates are among the genes upregulated by IL-4. How K^+^ and H^+^ secretion regulates apical pH, bicarbonate concentration in the airway surface fluid, and mucus dynamics will require future studies.

In conclusion, our study reveals the complexity of events triggered by IL-4. Since our experiments were done on cells from a small number of individuals, future studies will need to confirm these observations in different cell/animal models and *ex vivo* samples from patients with different lung diseases. Elucidation of molecular mechanisms underlying goblet cell hyperplasia may help to understand pathological alterations occurring in asthma, cystic fibrosis, and other chronic respiratory diseases characterized by mucus hypersecretion, thus leading to possible development of novel therapeutic strategies.

## Methods

### Cell culture

The procedures for isolation and culture of human bronchial epithelial cells were described in detail in a previous study^14^. Briefly, mainstem human bronchi, derived from CF and non-CF individuals undergoing lung transplant were dissected, washed, and incubated overnight at 4 °C in protease XIV solution. Epithelial cells were then detached mechanically, dissociated by trypsinization, and cultured in flasks in a serum-free medium (LHC9/RPMI 1640). After 4–5 passages, cells were seeded at high density (500,000/cm^2^) on Snapwell 3801 porous inserts. After 24 hours from seeding, the medium was switched to DMEM/F12 (1:1) plus 2% New Zealand fetal bovine serum (Life Technologies), hormones, and supplements^14^. The medium was replaced daily on both sides of permeable supports up to 8–10 days (liquid-liquid culture, LLC). Subsequently the apical medium was totally removed and the cells received nutrients only from the basolateral side (air-liquid culture, ALC). This condition favored a further differentiation of the epithelium. Cells were maintained under ALC for 3 weeks. Cells were obtained from two non-CF subjects (BE37: patient with pulmonary hypertension; BE63: patient with idiopathic pulmonary fibrosis) and three CF subjects (BE43, BE49, BE91: patients with F508del/F508del genotype). For CF cells, the LCH9/RPMI 1640 medium also contained in the first 4 days additional antibiotics to eradicate bacterial contamination. For this purpose the mixture of antibiotics (usually colistin, piperacillin, and tazobactam) and dosage were designed on the basis of the antibiogram of bacteria isolated from the most recent expectorate of the patient.

All procedures related to the use of human epithelial cells were carried out in accordance with the approved guidelines. In particular, the protocols to isolate, culture, store, and study bronchial epithelial cells from patients undergoing lung transplant was approved by the Ethical Committee of Gaslini Institute under the supervision of the Italian Ministry of Health. Written informed consent was obtained from all patients using a form that was also approved by the same Ethical Committee.

### Microarray analysis

For the analysis of gene expression, microarray hybridization experiments were performed on three separate preparations of bronchial epithelial cells (BE37) differentiated on Snapwell inserts and treated with and without IL-4 for 6, 12, 24, and 72 hours. Total RNA for each condition was used for hybridization to the Affymetrix GeneChip Human Genome 133A2 array using standard protocols as previously described^35^. Differentially expressed genes were detected by a Bayesian t-test method, Cyber-t^36^ particularly suited when the number of replicates is limited. The Benjamini-Hochberg procedure was used to calculate the False Discovery Rate (FDR). The thresholds used were FDR < 0.05 unless otherwise stated. Microarray data are publicly available (GEO Access Number: GSE78914).

### Short-circuit current recordings

Snapwell supports carrying differentiated bronchial epithelia were mounted in a vertical chamber resembling an Ussing system with internal fluid circulation. Both apical and basolateral hemichambers were filled with 5 ml of a Krebs bicarbonate solution containing (in mM): 126 NaCl, 0.38 KH_2_PO_4_, 2.13 K_2_HPO_4_, 1 MgSO_4_, 1 CaCl_2_, 24 NaHCO_3_, and 10 glucose. Both sides were continuously bubbled with a gas mixture containing 5% CO_2_ – 95% air and the temperature of the solution was kept at 37 °C. The transepithelial voltage was short-circuited with a voltage-clamp (DVC-1000, World Precision Instruments) connected to the apical and basolateral chambers via Ag/AgCl electrodes and agar bridges (1 M KCl in 1% agar). The offset between voltage electrodes and the fluid resistance were canceled before experiments. The short-circuit current was recorded with a PowerLab 4/25 (ADInstruments) analogical to digital converter connected to a Macintosh computer.

### Western blot

To evaluate TMEM16A, CFTR, and SLC26A4 (pendrin) protein expression, bronchial epithelial cells, treated with or without IL-4 for 72 hours, were lysed in RIPA 1X buffer (50 mM Tris-HCl pH 7.4, 150 mM NaCl, 1% Triton X-100, 0.5% sodium deoxycholate, 0.1% SDS) containing Complete Protease Inhibitor Cocktail (Roche, NJ). For CFTR and TMEM16A, we also used lysates from parental CFBE41o- cells. For SLC26A4, we used lysates from HEK-293 MRS cells grown to subconfluence on 60 mm-diameter dishes and transiently transfected with 10 μl of Lipofectamine 2000 and 4 μg of mammalian expression plasmid pcDNA 3.1 carrying the coding sequence of full lenght SLC26A4 protein. Protein concentration in lysates was quantified using the Quantum Protein Assay kit (Euroclone). Fifty μg of total lysates (10 μg for HEK-293 cells) were separated onto Criterion TGX precast Gels 4–15% (Biorad) and transferred to nitrocellulose membrane (Biorad) for western blotting with Trans-Blot Turbo system (Biorad). TMEM16A protein was immunodetected by a rabbit monoclonal antibody (SP31, Abcam) 1:500, followed by anti-rabbit HRP (Abcam) 1:10000. CFTR protein was immunodetected with mouse monoclonal anti-CFTR antibody (596, Cystic Fibrosis Foundation Therapeutics and University of North Carolina, Chapel Hill) 1:2000 followed by anti-mouse HRP (Abcam) 1:10000 secondary antibody. SLC26A4 protein was detected using mouse polyclonal Ab anti SLC26A4 (A01) Abnova 1:1000 and anti-mouse HRP (Abcam) 1:5000 as secondary antibody. Carbonic anhydrase 2 was detected using rabbit monoclonal Ab anti-CAII (EPR5195, Abcam) 1:1000 and anti-rabbit HRP (Abcam) 1:5000 as secondary antibody. Membranes were also stripped with the Strip Ablot stripping buffer (Euroclone) and incubated with the mouse monoclonal C464.8 (Merck Millipore) antibody against Na^+^/K^+^-ATPase β1 (1:6000) or with mouse monoclonal anti-GAPDH antibody clone 6C5 (Santa Cruz Biotech Inc.) 1:5000 followed by anti-mouse HRP-conjugated secondary antibody (Ab 97023, Abcam; 1:10000).

All antibodies were dissolved in 5% skimmed-milk in TBS-T. Protein bands were visualized using the Super Signal West Femto Substrate (Thermo Fisher Scientific Inc). Direct recording of the chemiluminescence was performed using the Molecular Imager ChemiDoc XRS System (Biorad).

### Immunofluorescence

Primary human bronchial epithelial cells on Snapwell permeable supports were fixed by adding 200 μl of 10% neutral buffered formalin (05-01005Q, Bio-Optica) to the apical side for 10 minutes at room temperature. After three washings in PBS, cells were processed for antigen retrieval with 10 mM citrate buffer pH = 6 heated to 95 °C in a microwave for 5 minutes. Samples were then cooled to room temperature in PBS and permeabilized with PBS-Triton X-100 0.3% for 5 minutes. After washing, cells were blocked with 1% bovine serum albumin (BSA) in PBS for 2 hours and then incubated overnight at 4 °C with 200 μl of primary antibodies diluted in PBS-BSA 1%. The following primary antibodies and dilutions were used: rabbit monoclonal anti-TMEM16A [SP31] (ab64085, Abcam) at 1:200, mouse IgG1 anti-CFTR (ab570, J.R. Riordan, University of North Carolina at Chapel Hill, and Cystic Fibrosis Foundation Therapeutics) at 1:250, mouse polyclonal anti-SLC26A4 (H00005172-A01, Abnova) at 1:200, rabbit polyclonal anti-SLC12A2 (HPA020130, Sigma-Aldrich) at 1:1000, rabbit monoclonal anti-CA2 [EPR5195] (ab124687, Abcam) at 1:500, mouse IgG1 anti-MUC5AC (NCL-HGM-45M1, Novocastra) at 1:100, mouse IgG2B anti-acetylated tubulin (7451, Sigma Aldrich) at 1:300, rabbit polyclonal anti-ATP12A (HPA039526, Sigma-Aldrich) at 1:400.

Following incubation with primary antibody, cells were rinsed three times in PBS and incubated with 200 μl of a solution of secondary Alexa Fluor conjugated antibodies (Invitrogen) diluted 1:200 in PBS-BSA 1% for 1 hour in the dark. After further 3 washes in PBS, the porous membrane carrying the cells was cut from the plastic support of the Snapwell, placed on microscope slides and mounted with Fluoroshield with 4′,6-diamidino-2-phenylindole (DAPI) (Sigma-Aldrich) to stain cell nuclei.

Confocal microscopy was performed using a laser scanning confocal microscope TCS SP8 (Leica Microsystems, Heidelberg, Germany). Image analysis was performed using Leica and ImageJ software.

### Intracellular pH (pH_i_) measurements

Measurements were performed using a pH-sensitive fluorescent probe, BCECF/AM (2′,7′-bis(2-carboxyethyl)-5(6)-carboxyfluorescein acetoxymethylester; Life Technologies) and the fluorescence intensity was recorded at excitation wavelengths of 500 and 440 nm and at emission wavelength of 535 nm by a computer controlled spectrofluorometer (Cary Eclipse Varian). Briefly, bronchial epithelial cells grown on permeable supports in polyethylene terephthalate (BD Falcon) at ALC conditions, and loaded with BCECF, were mounted in a cuvette which allowed independent perfusion of the apical and basolateral sides. The solution contained (in mM): 115 NaCl, 5 KCl, 25 NaHCO_3_, 1 MgCl_2_, 1 CaCl_2_, 10 D-glucose. Solution pH was adjusted to 7.4 by bubbling with 95% O_2_ and 5% CO_2_. For Cl^−^-free conditions, NaCl was substituted with sodium gluconate, CaCl_2_ with 6 mM calcium gluconate, and KCl with 2.5 mM K_2_SO_4_. Experiments were carried out at 37 °C.

Calibration of fluorescence ratio to pH_i_ was performed after each experiment using nigericin (10 μM) and potassium ions (150 mM) at various external pH values varying between 6.0 and 8.0 as previously reported^37^. The value of ∆pH_i_ was calculated as the difference between the maximum value reached upon the perfusion with Cl-free solution and the value obtained by averaging the last 20 data points recorded before perfusion with the Cl^−^-free solution.

### Apical fluid composition

Differentiated bronchial epithelia under ALC condition were treated for 72 hours with or without IL-4 (10 ng/ml). At 48 hours before the end of the experiment, 150 μl of Krebs solution (see short-circuit recordings paragraph) were pipetted on the apical side of epithelia. At the end, the fluid was recovered in a single step (i.e. without repeated pipetting) and ion composition was rapidly analyzed with a Cobas 6000 analyzer (Roche Diagnostics). To measure pH, we used litmus strips (6.0–8.1 and 7.2–8.8 range). The reading of the pH value was done by an operator blinded to the condition of the samples. To confirm these results, pH was also measured on a separate set of experiments using pH-sensitive microelectrodes (World Precision Instruments).

### Mucus release assay

Bronchial epithelia, differentiated on Snapwell permeable supports under ALC condition, were incubated for 3 hours with Coon’s modified Ham’s F12 medium (no serum) on the basolateral side. The medium was buffered with bicarbonate (2.68 g/L) or with 25 mM HEPES (pH = 7.2). After incubation, Snapwell supports were tilted by 10 degrees and 50 μL of PBS containing ATP 100 μM and yellow/green FluoSpheres beads of 200 nm diameter diluted 1:1000 (F8811, Life Technologies) were added to the apical side of epithelia just above the center of the permeable support. After 2 minutes, excess fluid was removed in a single step and snapwell supports were repositioned horizontally and transferred to a Nikon Eclipse TiE fluorescence microscope equipped with a 20x objective and GFP optical filter. An automated tile scan acquisition in the center of the permeable support (5 × 5 fields for a total surface of 5.8 mm^2^) was performed for each epithelium. The perfect focus system (PFS) was used to control the focal plane.

Image analysis was performed using Image J software implemented with a plug-in for texture analysis based on the Gray Level Co-occurrence Matrices method (GLCM texture). The GLCM for 0° and 90° and for a distance of 1 pixel were calculated and averaged. The following texture features were extracted: angular second moment, contrast, correlation, inverse difference moment and entropy.

### Data presentation and analysis

Results are shown as representative traces/images and as mean ± SEM. Significance was determined using Student’s t test or ANOVA with Tukey’s post-test using the InStat3 software.

## Additional Information

**How to cite this article**: Gorrieri, G. *et al.* Goblet Cell Hyperplasia Requires High Bicarbonate Transport To Support Mucin Release. *Sci. Rep.* **6**, 36016; doi: 10.1038/srep36016 (2016).

**Publisher's note**: Springer Nature remains neutral with regard to jurisdictional claims in published maps and institutional affiliations.

## Supplementary Material

Supplementary Information
