# Supplementary material for: Goblet Cell Hyperplasia Requires High Bicarbonate Transport To Support Mucin Release
Source: Sci Rep. 2016 Oct 27;6:36016. doi: 10.1038/srep36016 (PMC5081536; doi:10.1038/srep36016)
Supplement: Supplementary Information [file srep36016-s1.pdf]

Supplementary Material

GOBLET CELL HYPERPLASIA REQUIRES HIGH BICARBONATE TRANSPORT TO SUPPORT MUCIN RELEASE

Giulia Gorrieri, Paolo Scudieri, Emanuela Caci, Marco Schiavon, Valeria Tomati, Francesco Sirci, Francesco Napolitano, Diego Carrella, Ambra Gianotti, Ilaria Musante, Maria Favia, Valeria Casavola, Lorenzo Guerra, Federico Rea, Roberto Ravazzolo, Diego Di Bernardo, Luis J.V. Galietta

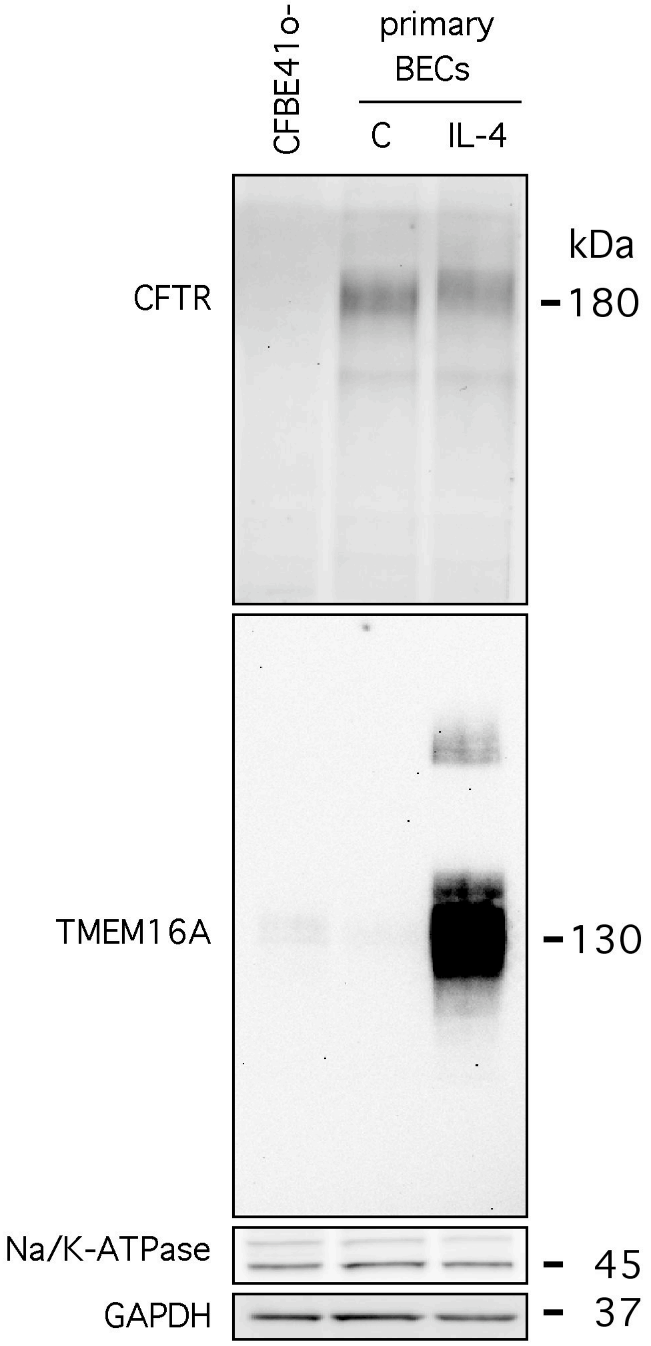

Supplementary Figure 1. Detection of CFTR and TMEM16A. The figure shows full-length western blots from the experiments presented in Figure 1.

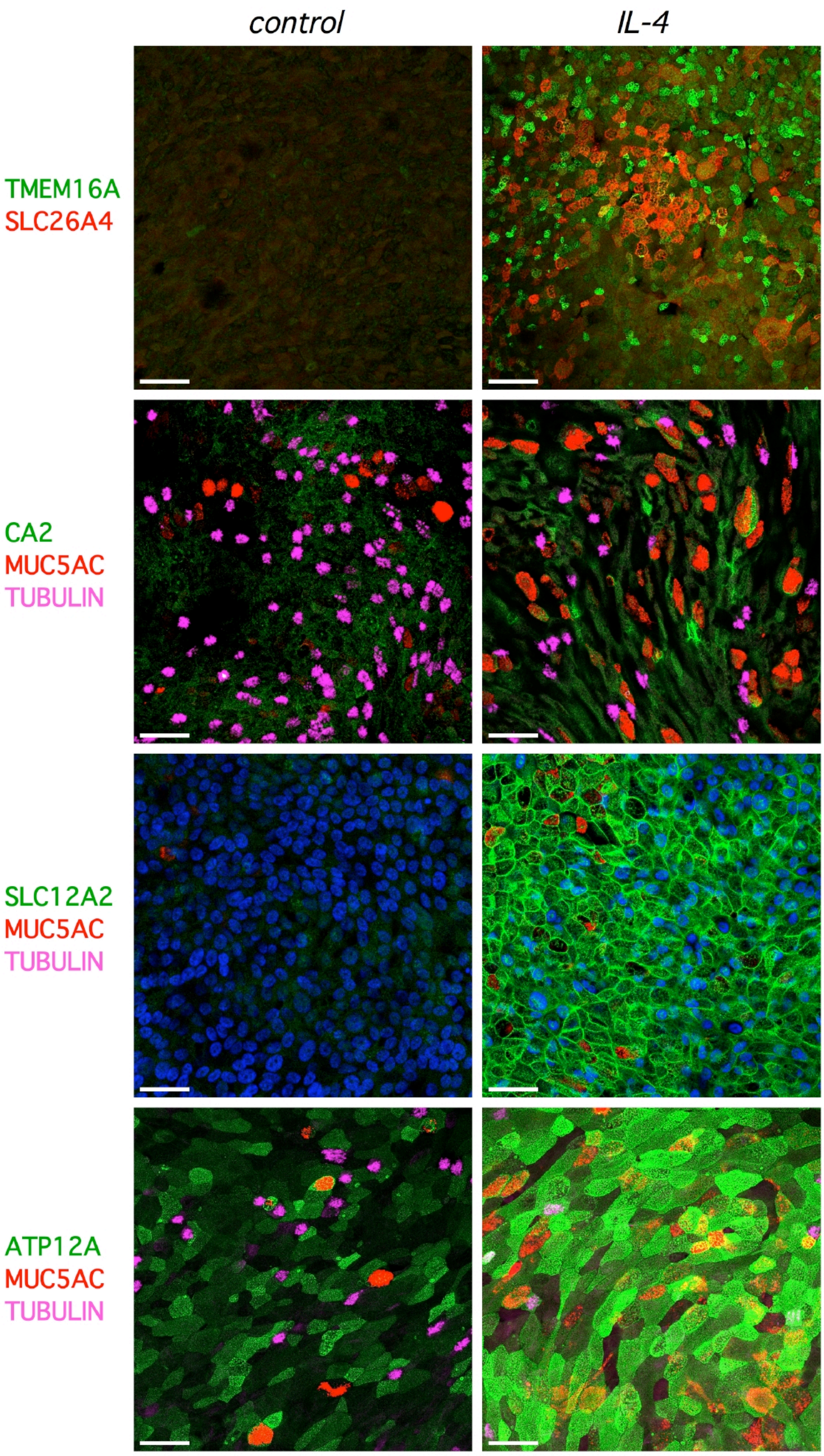

Supplementary Figure 2. Detection of proteins upregulated by IL-4. Representative confocal microscope images reporting localization and extent of expression of TMEM16A, SLC26A4, CA2, SLC12A2, and ATP12A in control and IL-4 treated cells. Where indicated, expression of cilia (tubulin) and MUC5AC is also shown. Images are of larger size (scale bar: 50 µm) with respect to those shown in Fig. 3. It should be noted that confocal SLC12A2 images were taken at a height closer to basal side. Therefore, MUC5AC, which is more localized towards the apical pole of cells, is less visible.

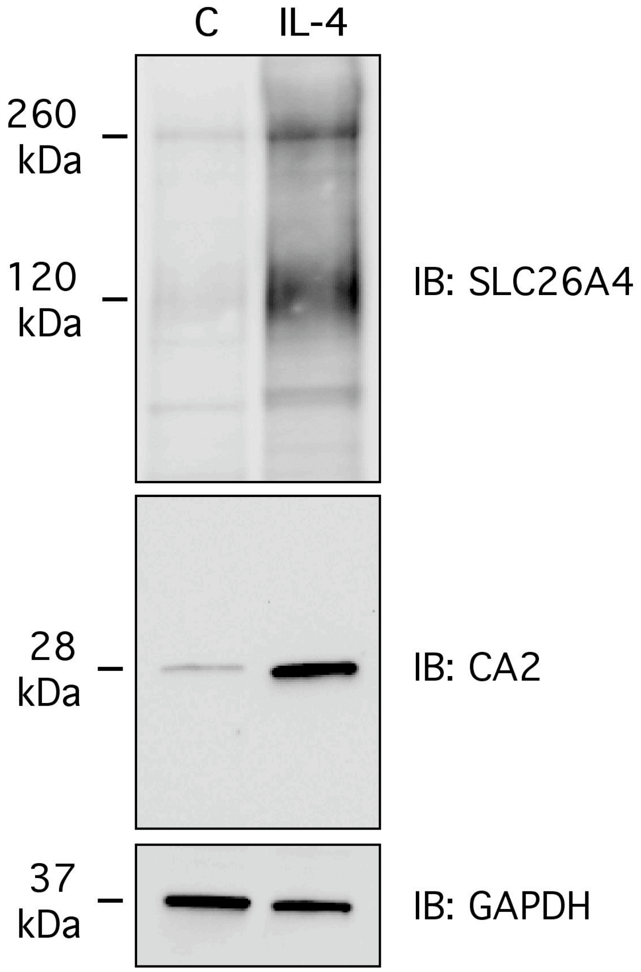

Supplementary Figure 3. Upregulation of SLC26A4 and carbonic anhydrase 2. Images show representative western blots to detect expression of SLC26A4 (pendrin), carbonic anhydrase 2 (CA2), and GAPDH in cells (BE37) treated with and without IL-4 for 72 hrs. The results are representative of three similar experiments.

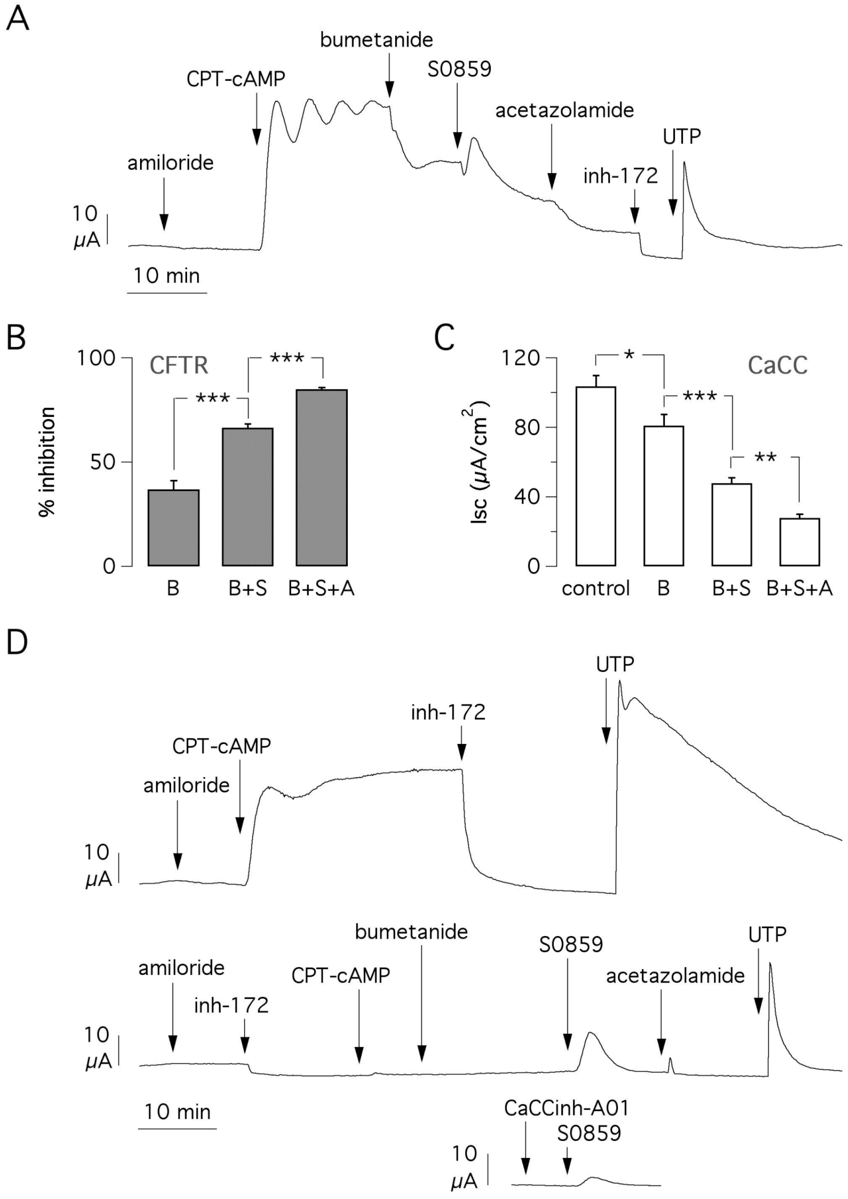

Supplementary Figure 4. Pharmacological inhibition of anion transport. (A) Representative trace showing additive effects caused by sequential addition of bumetanide, S0859, and acetazolamited on cells treated with IL-4 for 72 hrs. (B) Summary of inhibition caused by pharmacological modulators on CFTR-dependent secretion. Data are reported as % inhibition of total CFTR-dependent current. ***, $p < 0.001$ (n = 8; BE37 cells). (C) Summary of inhibition caused by pharmacological modulators on Ca^{2+}-activated secretion. Data are reported as the peak of UTP-dependent current measured in the various conditions. *, $p < 0.05$; **, $p < 0.01$; ***, $p < 0.001$ (n = 8; BE37 cells). (D) Pharmacological analysis of CFTR-dependent secretion. Prior addition of $CFTR_{inh}$-172 prevents the activation by CPT-cAMP and eliminates the inhibitory response to bumetanide, S0859, and acetazolamide. As shown in the inset, the transient activation elicited by S0859 was strongly inhibited by $CaCC_{inh}$-A01.

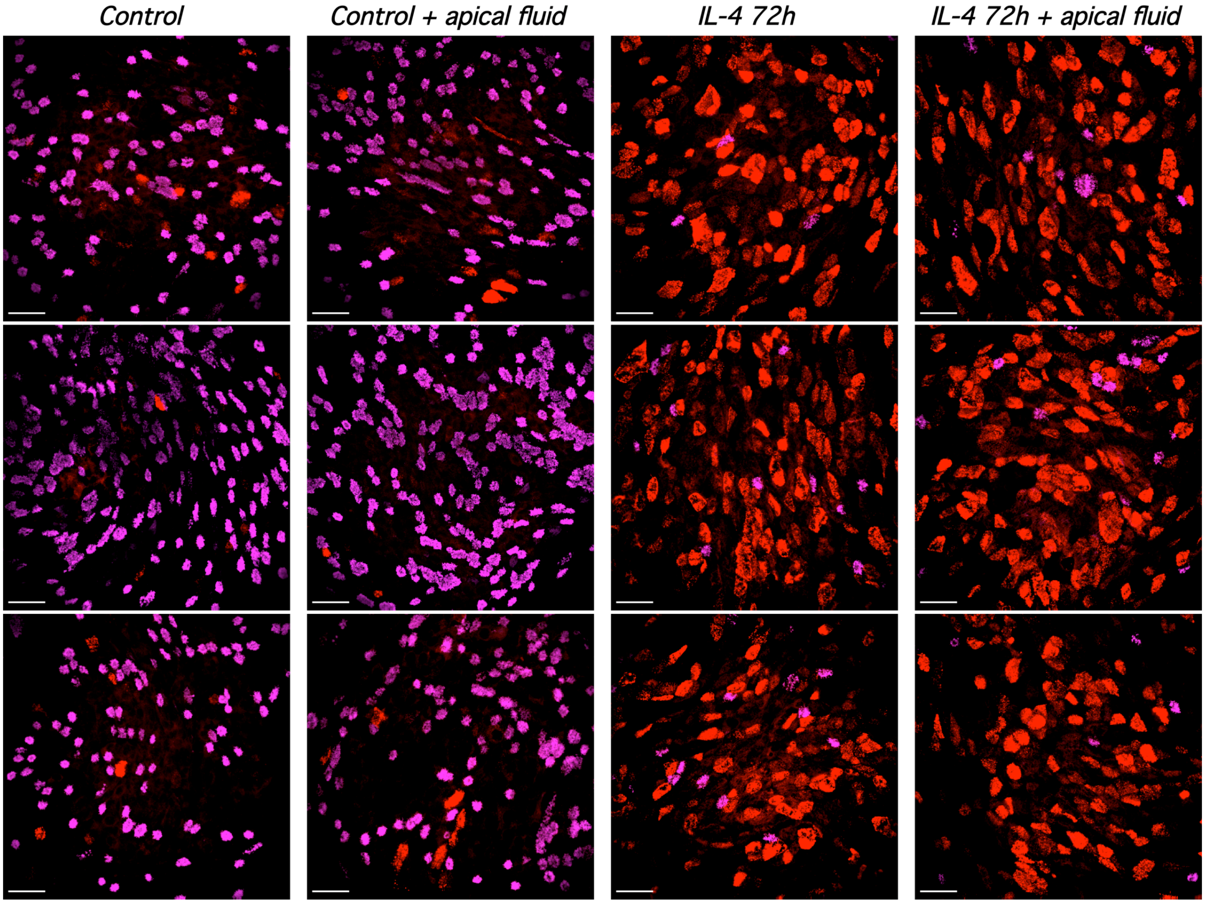

Supplementary Figure 5. Goblet cell hyperplasia in submerged cells. Representative images (three images per condition) showing detection of cilia (magenta) and MUC5AC (red) in cultured bronchial epithelia kept under air-liquid condition or after addition of fluid (150 μl) on the apical side (submerged condition) for 48 hours. As previously described (ref. 14), IL-4 (72 hours) markedly reduces the number of ciliated cells and increases the number of goblet cells. Scale bar: 50 μm.

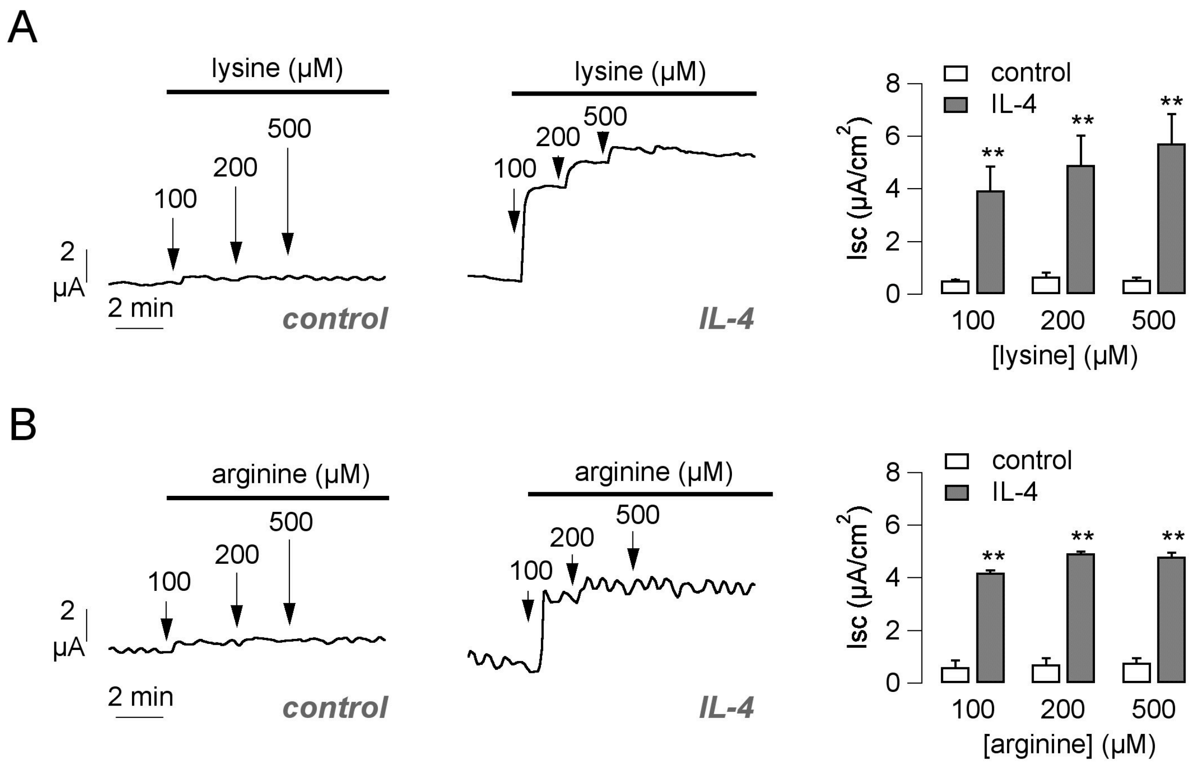

Supplementary Figure 6. Electrogenic amino acid transport. (A,B) Representative traces and bar graphs showing the dose-dependent increase in transepithelial current induced by apical application of lysine (A) or arginine (B). Experiments were done in the presence of amiloride (10 μM) and CFTR$_{inh}$-172 (10 μM). **, $p < 0.01$ vs untreated cells (n = 3; BE37 cells).

Supplementary Table 1

6 hours treatment with IL-4

	id_chip	*entrez_gene*	*signed_ratio*	*FDR*
1	210809_s_at	POSTN	53.13546009	4.67301E-05
2	209301_at	CA2	27.11271383	6.79594E-06
3	220622_at	LRRC31	25.12822349	3.09575E-06
4	206026_s_at	TNFAIP6	14.29279637	0.000494296
5	206172_at	IL13RA2	13.20023991	4.28889E-05
6	206432_at	HAS2	13.1506566	3.54774E-06
7	206932_at	CH25H	12.36677254	2.72265E-06
8	206262_at	ADH1C	11.72963485	1.39746E-06
9	207328_at	ALOX15	10.642624	9.71102E-05
10	210001_s_at	SOCS1	9.644127879	1.11438E-05
11	203913_s_at	HPGD	8.226463946	1.70158E-05
12	219764_at	FZD10	8.193579896	6.24785E-06
13	211478_s_at	DPP4	8.077645423	3.59425E-06
14	211548_s_at	HPGD	7.961116887	9.11163E-06
15	206529_x_at	SLC26A4	7.836767172	0.001414337
16	218804_at	ANO1	7.703179252	2.75805E-05
17	203914_x_at	HPGD	7.564008879	1.19641E-05
18	206025_s_at	TNFAIP6	7.2873543	0.000634773
19	205630_at	CRH	7.082005871	0.005483914
20	203717_at	DPP4	6.571797411	5.72509E-05
21	211549_s_at	HPGD	6.350015926	0.000185858
22	219564_at	KCNJ16	6.006725542	0.002377799
23	214981_at	POSTN	5.9506005	0.052571872
24	219334_s_at	OBFC2A	5.758603472	4.66015E-05
25	208350_at	CSN1S1	5.570472478	0.020323331
26	212942_s_at	KIAA1199	5.547756014	2.14352E-05
27	204614_at	SERPINB2	5.523381276	9.47168E-05
28	203434_s_at	MME	5.500197391	0.000278061
29	221223_x_at	CISH	5.428768459	4.00736E-06
30	208605_s_at	NTRK1	5.270939949	0.000140256
31	219728_at	MYOT	5.089773063	2.35105E-05
32	214539_at	SERPINB10	4.909537478	7.68583E-06
33	204463_s_at	EDNRA	4.831141201	8.1754E-05
34	205880_at	PRKD1	4.559101436	0.000287812
35	219508_at	GCNT3	4.435722502	3.91045E-05
36	219531_at	CEP72	4.426988021	5.82603E-06
37	202237_at	NNMT	4.425032085	0.000246407
38	206070_s_at	EPHA3	4.3790527	0.006955423
39	209267_s_at	SLC39A8	4.327213482	6.20622E-06
40	213880_at	LGR5	4.317519946	0.000522132
41	219619_at	DIRAS2	4.215626724	0.000165577
42	207820_at	ADH1A	4.112738819	0.000848415
43	207414_s_at	PCSK6	4.070948248	2.71858E-06
44	212909_at	LYPD1	3.929813815	1.0178E-05
45	203716_s_at	DPP4	3.895226585	5.24077E-05
46	205466_s_at	HS3ST1	3.855214447	1.45083E-06
47	219142_at	RASL11B	3.854061666	5.20735E-05
48	219869_s_at	SLC39A8	3.800009409	0.00022389

49	204464_s_at	EDNRA	3.779092898	4.20919E-06
50	220745_at	IL19	3.700480982	5.69463E-05
51	205952_at	KCNK3	3.69971649	0.007071631
52	212741_at	MAOA	3.688926012	1.0063E-05
53	209340_at	UAP1	3.678817285	3.02218E-06
54	204388_s_at	MAOA	3.590477402	4.29753E-05
55	202238_s_at	NNMT	3.509244098	7.35174E-05
56	204389_at	MAOA	3.451533088	0.000148257
57	220192_x_at	SPDEF	3.359506613	7.49175E-06
58	206382_s_at	BDNF	3.312749282	0.000617586
59	210118_s_at	IL1A	3.28418073	4.99825E-06
60	57588_at	SLC24A3	3.284100752	0.000116209
61	204121_at	GADD45G	3.250189281	0.003917743
62	209955_s_at	FAP	3.206492161	0.001318688
63	214404_x_at	SPDEF	3.174037107	1.05816E-05
64	201925_s_at	CD55	3.162111965	4.75228E-06
65	205818_at	DBC1	3.093278912	4.20145E-05
66	219090_at	SLC24A3	2.96807723	5.73062E-05
67	216504_s_at	SLC39A8	2.919161387	0.000115865
68	217523_at	CD44	2.916843736	3.24076E-05
69	203650_at	PROCR	2.864718703	0.000120812
70	206942_s_at	PMCH	2.816307465	6.85833E-05
71	219648_at	MREG	2.79547826	6.62305E-05
72	206343_s_at	NRG1	2.789565343	3.6386E-05
73	219734_at	SIDT1	2.751450073	2.07555E-05
74	212158_at	SDC2	2.723960177	0.000204235
75	204491_at	PDE4D	2.718956238	5.30891E-05
76	205206_at	KAL1	2.710081008	5.16923E-05
77	203980_at	FABP4	2.690086471	0.003049933
78	218510_x_at	FAM134B	2.685441219	0.003473052
79	213106_at	ATP8A1	2.658903316	7.46147E-05
80	213441_x_at	SPDEF	2.629474787	6.78308E-05
81	218532_s_at	FAM134B	2.60155365	0.000904523
82	209606_at	CYTIP	2.588530058	0.003082736
83	216005_at	TNC	2.574417093	0.000165949
84	203435_s_at	MME	2.548051711	0.001308149
85	210139_s_at	PMP22	2.546510619	0.000475381
86	211906_s_at	SERPINB4	2.500861062	0.000165821
87	212944_at	SLC5A3	2.49057321	1.77842E-05
88	216258_s_at	SERPINB13	2.489570555	0.000387256
89	204041_at	MAOB	2.476538916	0.000933419
90	210314_x_at	TNFSF13	2.466448329	3.35144E-05
91	220317_at	LRAT	2.459072449	0.001215036
92	205876_at	LIFR	2.450664579	0.000481694
93	213164_at	SLC5A3	2.447468976	2.37603E-05
94	204337_at	RGS4	2.442296967	0.013880341
95	220780_at	PLA2G3	2.441548438	0.000896027
96	213107_at	TNIK	2.436115395	2.66977E-05
97	219987_at	LOC10028841[illegible]	2.432573152	0.001043184
98	205579_at	HRH1	2.385179778	4.31506E-05
99	202207_at	ARL4C	2.37368889	2.99587E-05
100	211361_s_at	SERPINB13	2.362894918	0.000312444
101	206060_s_at	PTPN22	2.347521467	0.001384574

102	207526_s_at	IL1RL1	2.344758174	0.004186207
103	219836_at	ZBED2	2.344017493	5.70911E-05
104	214787_at	DENND4A	2.340657672	4.73787E-05
105	206756_at	CHST7	2.338029298	0.00010757
106	211362_s_at	SERPINB13	2.330005559	0.000237464
107	215446_s_at	LOX	2.299547841	2.97065E-05
108	205185_at	SPINK5	2.29912994	0.008084906
109	206135_at	ST18	2.29765354	0.002196061
110	202437_s_at	CYP1B1	2.296632557	0.016264795
111	205381_at	LRRC17	2.287645633	0.000353853
112	205190_at	PLS1	2.284632377	1.71895E-05
113	220260_at	TBC1D19	2.269508616	5.15701E-05
114	209500_x_at	TNFSF12-TNFS	2.263152142	2.77678E-05
115	205214_at	STK17B	2.262695173	0.001005117
116	218935_at	EHD3	2.261245252	0.000218586
117	219727_at	DUOX2	2.256052061	6.83992E-05
118	205027_s_at	MAP3K8	2.25215713	0.001747947
119	211828_s_at	TNIK	2.252123483	0.00038298
120	202134_s_at	WWTR1	2.250308068	0.002844638
121	202037_s_at	SFRP1	2.236288742	0.000598974
122	201109_s_at	THBS1	2.231682274	0.00105969
123	205590_at	RASGRP1	2.208311046	0.015763959
124	202206_at	ARL4C	2.202447875	9.44575E-05
125	209499_x_at	TNFSF12-TNFS	2.20148989	7.06438E-05
126	209930_s_at	NFE2	2.190665072	0.000510352
127	202436_s_at	CYP1B1	2.188575063	0.009261461
128	203560_at	GGH	2.16342202	0.000809138
129	220049_s_at	PDCD1LG2	2.161550953	0.003049266
130	217272_s_at	SERPINB13	2.155850893	0.000507756
131	205969_at	AADAC	2.152793425	0.002251521
132	211495_x_at	TNFSF12-TNFS	2.136928034	0.00022948
133	220266_s_at	KLF4	2.134662387	0.001359804
134	218854_at	DSE	2.125594831	0.000237399
135	213721_at	SOX2	2.11584369	9.86753E-05
136	218943_s_at	DDX58	2.110981483	0.033983277
137	203058_s_at	PAPSS2	2.110270059	0.000295924
138	212192_at	KCTD12	2.10761437	0.000627363
139	202435_s_at	CYP1B1	2.103984806	0.004599292
140	203060_s_at	PAPSS2	2.096505979	9.31757E-05
141	212154_at	SDC2	2.092843033	0.000357159
142	209747_at	TGFB3	2.088957027	9.43863E-05
143	214068_at	BEAN1	2.084457426	0.001007102
144	201926_s_at	CD55	2.078613862	0.000319209
145	204341_at	TRIM16	2.070566616	0.000254845
146	221011_s_at	LBH	2.052539574	0.000349178
147	205267_at	POU2AF1	2.048360972	0.00035861
148	202208_s_at	ARL4C	2.047481021	0.000404643
149	212188_at	KCTD12	2.038031919	0.00035382
150	203821_at	HBEGF	2.035040787	0.000181377
151	221841_s_at	KLF4	2.032793143	0.001332142
152	218886_at	PAK1IP1	2.032062896	0.000128839
153	216235_s_at	EDNRA	2.029128636	0.003518849
154	46323_at	CANT1	2.028805811	0.000413163

155	204126_s_at	CDC45	2.018608066	0.002161849
156	213172_at	TTC9	2.016220702	8.16867E-05
157	205465_x_at	HS3ST1	2.009392356	0.000724332
158	205501_at	PDE10A	2.00526763	0.010829266
159	203759_at	ST3GAL4	1.99043285	0.000808584
160	205180_s_at	ADAM8	1.987845964	0.000876112
161	211020_at	GCNT2	1.97442318	0.000974734
162	215719_x_at	FAS	1.971712468	0.001200084
163	38037_at	HBEGF	1.968956581	0.000917207
164	204404_at	SLC12A2	1.968102483	0.00031212
165	214014_at	CDC42EP2	1.966583321	0.000253966
166	205192_at	MAP3K14	1.966041959	0.002591454
167	210553_x_at	PCSK6	1.963798724	0.001195554
168	209602_s_at	GATA3	1.960854472	0.00805965
169	213109_at	TNIK	1.95999037	0.000839542
170	212976_at	LRRC8B	1.95405778	0.006938914
171	221732_at	CANT1	1.953830581	9.72993E-05
172	204298_s_at	LOX	1.950243495	0.00036819
173	207455_at	P2RY1	1.947607843	0.003790792
174	220948_s_at	ATP1A1	1.947304705	2.24174E-05
175	204475_at	MMP1	1.93779331	0.003281271
176	220121_at	LINS	1.92698651	0.011540939
177	205532_s_at	CDH6	1.922591909	0.000417624
178	205533_s_at	CDH6	1.920406659	0.000575025
179	201981_at	PAPPA	1.900789746	0.001234766
180	212565_at	STK38L	1.900705741	0.001528836
181	202079_s_at	TRAK1	1.893236118	0.002001585
182	204352_at	TRAF5	1.887408512	0.001787432
183	204589_at	NUAK1	1.886893182	0.001003659
184	204818_at	HSD17B2	1.886718489	0.000715481
185	220658_s_at	ARNTL2	1.869846618	0.000328468
186	205273_s_at	PITRM1	1.869129034	0.000270684
187	206354_at	SLCO1B3	1.857204873	0.003011714
188	216598_s_at	CCL2	1.856608727	0.026081674
189	212196_at	IL6ST	1.856275409	0.00015214
190	204863_s_at	IL6ST	1.85531555	0.001415216
191	202236_s_at	SLC16A1	1.849820919	0.000713667
192	219630_at	PDZK1IP1	1.843796072	0.000218293
193	214621_at	GYS2	1.842285306	0.002549529
194	218684_at	LRRC8D	1.83948326	0.000185949
195	216252_x_at	FAS	1.832607812	0.003920129
196	36564_at	RNF19B	1.8324646	0.00100931
197	209604_s_at	GATA3	1.832281347	0.000617147
198	212572_at	STK38L	1.830818099	0.004095531
199	205042_at	GNE	1.830118855	0.000460277
200	221840_at	PTPRE	1.823111148	0.00142337

Supplementary Table 2

12 hours treatment with IL-4

	id_chip	*entrez_gene*	*signed_ratio*	*FDR*
1	210809_s_at	POSTN	161.7079419	0.000206453
2	209301_at	CA2	25.50883151	7.36537E-06
3	207328_at	ALOX15	25.49549221	0.000122393
4	220622_at	LRRC31	25.41418488	8.86395E-07
5	206932_at	CH25H	18.70160447	1.13191E-05
6	211478_s_at	DPP4	16.5750696	1.00565E-06
7	206172_at	IL13RA2	15.48827622	6.1581E-05
8	206262_at	ADH1C	14.67205061	0.000277224
9	214539_at	SERPINB10	14.22621466	8.77264E-07
10	206529_x_at	SLC26A4	12.49880914	0.002983057
11	208605_s_at	NTRK1	12.06883776	1.62213E-05
12	208350_at	CSN1S1	11.95164317	0.000756751
13	203717_at	DPP4	11.58120395	6.99255E-06
14	203716_s_at	DPP4	10.00833935	1.39756E-05
15	203913_s_at	HPGD	9.447743228	7.16489E-07
16	219764_at	FZD10	9.409627848	8.98365E-07
17	218804_at	ANO1	9.005955725	9.65086E-05
18	206343_s_at	NRG1	8.677864748	2.5985E-06
19	205630_at	CRH	8.129608516	0.001464871
20	211548_s_at	HPGD	7.897305959	9.80942E-07
21	203914_x_at	HPGD	7.817075163	2.22559E-06
22	203434_s_at	MME	7.664059345	2.40319E-06
23	211549_s_at	HPGD	7.617036182	1.72399E-06
24	209267_s_at	SLC39A8	7.520316485	8.18253E-07
25	218585_s_at	DTL	7.163272666	0.017605138
26	206432_at	HAS2	7.062890991	0.002320864
27	219334_s_at	OBFC2A	6.470853177	1.82168E-05
28	219564_at	KCNJ16	6.317757775	8.52851E-06
29	212942_s_at	KIAA1199	6.200055931	5.48171E-05
30	219869_s_at	SLC39A8	6.160263443	5.59933E-06
31	210001_s_at	SOCS1	5.926521388	2.6913E-06
32	219619_at	DIRAS2	5.56085629	0.000413092
33	205034_at	CCNE2	5.438308216	0.002901046
34	206382_s_at	BDNF	5.38541114	1.09659E-05
35	219728_at	MYOT	5.270847294	1.65032E-06
36	209955_s_at	FAP	4.960418332	0.000122292
37	204614_at	SERPINB2	4.918603494	1.13703E-06
38	219142_at	RASL11B	4.899128842	5.39046E-05
39	221223_x_at	CISH	4.862075814	1.75875E-06
40	219508_at	GCNT3	4.802656709	0.000182938
41	211122_s_at	CXCL11	4.788108872	0.470407367
42	206026_s_at	TNFAIP6	4.665152741	0.000738067
43	209773_s_at	RRM2	4.51061735	0.176059455
44	209340_at	UAP1	4.505040088	4.1758E-06
45	206942_s_at	PMCH	4.492824871	0.000128434
46	204464_s_at	EDNRA	4.449098938	4.37352E-05
47	216504_s_at	SLC39A8	4.438677715	0.000114179
48	203153_at	IFIT1	4.400353	0.162730072

49	210521_s_at	FETUB	4.398125222	9.48071E-05
50	57588_at	SLC24A3	4.321759217	0.000122486
51	212909_at	LYPD1	4.234099855	7.9953E-05
52	204463_s_at	EDNRA	4.16812373	4.8487E-06
53	219090_at	SLC24A3	4.16783532	0.000113182
54	214981_at	POSTN	4.149640933	7.28569E-05
55	201890_at	RRM2	4.067560207	0.117262696
56	219836_at	ZBED2	4.016580278	6.18568E-05
57	203650_at	PROCR	3.984136988	0.000347102
58	202237_at	NNMT	3.981625022	5.12537E-05
59	205466_s_at	HS3ST1	3.906747859	2.38397E-05
60	203435_s_at	MME	3.885947304	3.34547E-05
61	212158_at	SDC2	3.811571209	0.000358237
62	203980_at	FABP4	3.789928479	0.206789748
63	212154_at	SDC2	3.738142919	0.002018764
64	211906_s_at	SERPINB4	3.720461863	1.13059E-05
65	204121_at	GADD45G	3.547201879	0.000306764
66	210163_at	CXCL11	3.499037409	0.382500119
67	204388_s_at	MAOA	3.472312534	3.75327E-05
68	206354_at	SLCO1B3	3.437770141	1.0709E-05
69	204389_at	MAOA	3.428348534	8.95547E-05
70	220192_x_at	SPDEF	3.42098703	3.81324E-05
71	219531_at	CEP72	3.381531004	1.48001E-05
72	206070_s_at	EPHA3	3.379188946	0.002784962
73	220780_at	PLA2G3	3.36346336	0.001576505
74	219727_at	DUOX2	3.341836425	0.000851814
75	220651_s_at	MCM10	3.331924083	0.012730563
76	220745_at	IL19	3.313021148	0.000269422
77	216258_s_at	SERPINB13	3.283144668	7.8153E-06
78	205969_at	AADAC	3.275656122	1.59258E-05
79	207414_s_at	PCSK6	3.257355057	4.75976E-06
80	203290_at	HLA-DQA1	3.226034423	0.387987776
81	203560_at	GGH	3.217168197	7.7579E-06
82	204415_at	IFI6	3.213167001	0.081177454
83	201925_s_at	CD55	3.199792263	0.000104582
84	210314_x_at	TNFSF13	3.138444808	6.1835E-05
85	202411_at	IFI27	3.113120803	0.006612317
86	209606_at	CYTIP	3.108183862	0.001205805
87	214404_x_at	SPDEF	3.105399242	3.25898E-05
88	205880_at	PRKD1	3.056285968	0.000162333
89	209500_x_at	TNFSF12-TNFS	3.03893323	7.2452E-05
90	207820_at	ADH1A	3.032142864	0.000102693
91	204491_at	PDE4D	2.99620754	0.000944034
92	209499_x_at	TNFSF12-TNFS	2.980894376	6.85609E-05
93	212741_at	MAOA	2.969838475	0.00048956
94	205818_at	DBC1	2.954596008	5.47073E-05
95	210118_s_at	IL1A	2.923701424	3.19953E-05
96	207367_at	ATP12A	2.919893729	0.02864551
97	213441_x_at	SPDEF	2.902932526	0.000117774
98	206835_at	STATH	2.895595633	0.000101467
99	205952_at	KCNK3	2.887356096	0.014837513
100	203967_at	CDC6	2.882434933	0.000305282
101	205190_at	PLS1	2.882370735	5.98085E-06

102	213721_at	SOX2	2.858229791	0.000329112
103	206025_s_at	TNFAIP6	2.850368205	0.004137169
104	207526_s_at	IL1RL1	2.827084015	0.080052266
105	217523_at	CD44	2.80574532	7.76121E-05
106	202037_s_at	SFRP1	2.770540882	4.34097E-05
107	214068_at	BEAN1	2.738790847	0.001311452
108	205381_at	LRRC17	2.669087286	8.30964E-05
109	219795_at	SLC6A14	2.649377806	5.99406E-06
110	208126_s_at	CYP2C18	2.633622833	0.0016167
111	213975_s_at	LYZ	2.632619712	0.011885234
112	219148_at	PBK	2.621361335	0.034309666
113	211495_x_at	TNFSF12-TNFS	2.62107117	0.000166006
114	202238_s_at	NNMT	2.618271367	9.13006E-05
115	218532_s_at	FAM134B	2.609287825	8.31529E-05
116	211362_s_at	SERPINB13	2.597469714	5.18141E-05
117	219493_at	SHCBP1	2.59698135	8.16044E-05
118	206693_at	IL7	2.588525601	0.000828554
119	211990_at	HLA-DPA1	2.583359434	0.175087779
120	215103_at	CYP2C18	2.579022754	0.000674826
121	204529_s_at	TOX	2.571926335	0.007264702
122	218935_at	EHD3	2.567741236	0.000357964
123	201292_at	TOP2A	2.545320154	0.300800504
124	201627_s_at	INSIG1	2.544174609	0.016397748
125	205206_at	KAL1	2.523125912	0.000164071
126	204114_at	NID2	2.516635305	0.000674782
127	218943_s_at	DDX58	2.490410361	0.000358151
128	204126_s_at	CDC45	2.483266858	3.59535E-05
129	210559_s_at	CDK1	2.477435395	0.063935322
130	201291_s_at	TOP2A	2.469110325	0.339559333
131	214787_at	DENND4A	2.458303329	5.09484E-05
132	204146_at	RAD51AP1	2.458272122	0.000550238
133	204404_at	SLC12A2	2.452242786	0.00027305
134	209875_s_at	SPP1	2.447669787	0.006907511
135	211991_s_at	HLA-DPA1	2.426772175	0.230945521
136	205860_x_at	FOLH1	2.420867047	0.000544956
137	211814_s_at	CCNE2	2.420428263	0.000340266
138	218510_x_at	FAM134B	2.415554296	0.00011102
139	208510_s_at	PPARG	2.414240139	9.04041E-05
140	201626_at	INSIG1	2.41167118	0.012104875
141	220658_s_at	ARNTL2	2.408466978	6.11199E-05
142	221011_s_at	LBH	2.406530253	0.001851363
143	218681_s_at	SDF2L1	2.406114295	2.7477E-05
144	213537_at	HLA-DPA1	2.396351222	0.065467074
145	203968_s_at	CDC6	2.388439541	0.000127135
146	205890_s_at	GABBR1	2.385552752	6.19799E-05
147	204341_at	TRIM16	2.381883356	0.000321848
148	220133_at	ODAM	2.376787091	0.003977367
149	203213_at	CDK1	2.375308974	0.100889117
150	205533_s_at	CDH6	2.356781701	0.000100129
151	214452_at	BCAT1	2.350893367	0.000722237
152	205027_s_at	MAP3K8	2.347893654	6.2341E-05
153	201926_s_at	CD55	2.344559413	0.001724002
154	211361_s_at	SERPINB13	2.335292033	1.99366E-05

155	205185_at	SPINK5	2.324107842	7.21273E-05
156	204929_s_at	VAMP5	2.320156295	4.40018E-05
157	220260_at	TBC1D19	2.312188166	3.82029E-05
158	205909_at	POLE2	2.309748163	0.004376949
159	206756_at	CHST7	2.307332181	0.000100828
160	221841_s_at	KLF4	2.30586912	0.000211269
161	209930_s_at	NFE2	2.303072653	0.000336319
162	221060_s_at	TLR4	2.291814488	0.000305001
163	202206_at	ARL4C	2.289964504	9.11182E-05
164	219429_at	FA2H	2.288235764	0.0018171
165	213880_at	LGR5	2.285696061	0.001607956
166	218886_at	PAK1IP1	2.284179058	5.37115E-05
167	205532_s_at	CDH6	2.281858323	0.000203289
168	212157_at	SDC2	2.281133039	0.000743492
169	219987_at	LOC10028841	2.278751327	0.000127961
170	205968_at	KCNS3	2.272206765	0.000548448
171	205084_at	BCAP29	2.272102359	0.000102439
172	215363_x_at	FOLH1	2.261366504	0.000913182
173	217272_s_at	SERPINB13	2.257377696	1.06642E-05
174	213107_at	TNIK	2.251211644	8.3084E-05
175	219684_at	RTP4	2.248358218	0.001174673
176	210602_s_at	CDH6	2.227793486	0.000421133
177	219983_at	HRASLS	2.226961662	0.000133836
178	221521_s_at	GINS2	2.222220289	0.006125894
179	205214_at	STK17B	2.221947032	7.24652E-05
180	202207_at	ARL4C	2.219010557	4.80756E-05
181	210004_at	OLR1	2.212261909	0.000220168
182	219258_at	TIPIN	2.209542565	0.000205964
183	205024_s_at	RAD51	2.207455643	0.028853779
184	220266_s_at	KLF4	2.202944459	0.000300815
185	204475_at	MMP1	2.201500086	0.002604205
186	218662_s_at	NCAPG	2.193717688	0.042421899
187	200973_s_at	TSPAN3	2.189822947	7.82843E-05
188	220049_s_at	PDCD1LG2	2.183699097	0.000229513
189	213106_at	ATP8A1	2.182901746	0.000195759
190	201625_s_at	INSIG1	2.181512972	0.063748474
191	205590_at	RASGRP1	2.180776933	0.002018919
192	202201_at	BLVRB	2.176753572	8.17424E-05
193	220446_s_at	CHST4	2.171464692	0.003629351
194	207165_at	HMMR	2.167799725	0.111889238
195	205042_at	GNE	2.163037025	0.000203385
196	201930_at	MCM6	2.162061748	0.002087467
197	204026_s_at	ZWINT	2.152593352	0.000908423
198	206391_at	RARRES1	2.143550479	0.000228216
199	205379_at	CBR3	2.140465097	0.00027992
200	210305_at	PDE4DIP	2.126649713	0.000217902

Supplementary Table 3

24 hours treatment with IL-4

	id_chip	*entrez_gene*	*signed_ratio*	*FDR*
1	210809_s_at	POSTN	298.2118122	2.09835E-05
2	220622_at	LRRC31	47.1807145	3.09147E-06
3	214539_at	SERPINB10	25.53323975	0.000232669
4	210521_s_at	FETUB	23.53432606	0.000321173
5	211478_s_at	DPP4	20.43143859	4.40387E-06
6	209301_at	CA2	20.11999037	1.47462E-05
7	218804_at	ANO1	18.24175809	2.89208E-05
8	206932_at	CH25H	15.62187254	2.56301E-06
9	207328_at	ALOX15	15.15914251	4.08263E-06
10	203717_at	DPP4	15.11233556	4.08277E-06
11	206529_x_at	SLC26A4	14.8364986	1.92083E-06
12	203716_s_at	DPP4	13.19121532	1.7552E-06
13	206343_s_at	NRG1	13.13982073	2.48674E-06
14	206432_at	HAS2	11.69408438	1.36559E-05
15	208605_s_at	NTRK1	11.14286958	1.87319E-05
16	203434_s_at	MME	11.0535531	9.26357E-07
17	206942_s_at	PMCH	10.35443331	0.000126439
18	203913_s_at	HPGD	9.492079693	3.12462E-06
19	211548_s_at	HPGD	8.655828045	2.32525E-06
20	204614_at	SERPINB2	8.228217019	0.000130572
21	203914_x_at	HPGD	8.211079771	2.1742E-06
22	211549_s_at	HPGD	7.914807719	1.01867E-05
23	209267_s_at	SLC39A8	7.829019207	2.0223E-05
24	219869_s_at	SLC39A8	7.523348121	1.42804E-05
25	219764_at	FZD10	7.200166618	2.10773E-06
26	205860_x_at	FOLH1	7.178844679	0.00045755
27	206382_s_at	BDNF	7.137004983	0.000971797
28	203650_at	PROCR	7.013228183	0.000405079
29	215363_x_at	FOLH1	6.779430188	0.004504382
30	219564_at	KCNJ16	6.313797134	0.000190734
31	206224_at	CST1	6.259539407	0.009800355
32	209606_at	CYTIP	6.085882624	0.000249704
33	210001_s_at	SOCS1	6.037840242	1.90656E-05
34	212942_s_at	KIAA1199	6.026552777	1.51084E-05
35	205969_at	AADAC	5.822510445	0.002267034
36	204121_at	GADD45G	5.795555661	0.002473959
37	209955_s_at	FAP	5.682450057	0.000551739
38	206262_at	ADH1C	5.370923415	0.000186152
39	211906_s_at	SERPINB4	5.216959711	9.45804E-06
40	203435_s_at	MME	5.170319574	2.7688E-05
41	219619_at	DIRAS2	4.931160318	0.011143598
42	219728_at	MYOT	4.874209712	0.002075538
43	208126_s_at	CYP2C18	4.842995222	4.0322E-05
44	208350_at	CSN1S1	4.825172691	0.003308302
45	219508_at	GCNT3	4.698663184	0.000500005
46	220192_x_at	SPDEF	4.662048425	4.00111E-05
47	219142_at	RASL11B	4.588716982	2.03063E-05
48	219334_s_at	OBFC2A	4.577525927	8.55175E-05

49	219727_at	DUOX2	4.573504756	0.000688051
50	206172_at	IL13RA2	4.495591407	0.05613616
51	201859_at	SRGN	4.468586247	0.000707588
52	219918_s_at	ASPM	4.380605835	0.00018976
53	214404_x_at	SPDEF	4.325290369	4.06564E-05
54	212158_at	SDC2	4.309853413	0.007216761
55	216504_s_at	SLC39A8	4.282145558	0.000174594
56	214068_at	BEAN1	4.250704479	0.001800996
57	204388_s_at	MAOA	4.218528373	1.79755E-05
58	221223_x_at	CISH	4.20533446	0.000106415
59	205630_at	CRH	4.199993117	0.000107245
60	218542_at	CEP55	4.192004086	0.000108275
61	204389_at	MAOA	4.182063409	0.000118097
62	209340_at	UAP1	4.164442461	6.46043E-06
63	219148_at	PBK	4.149305297	0.000321138
64	215103_at	CYP2C18	4.146284417	2.31841E-05
65	220745_at	IL19	4.139325951	0.001258039
66	214981_at	POSTN	4.135635757	0.000487982
67	207367_at	ATP12A	4.091974106	0.00015561
68	203980_at	FABP4	4.058694081	0.120453622
69	216258_s_at	SERPINB13	4.053803894	0.00012995
70	209555_s_at	CD36	3.985305716	0.056630961
71	212154_at	SDC2	3.977291947	0.005789269
72	213974_at	ADAMTSL3	3.967016657	0.000813977
73	57588_at	SLC24A3	3.911328808	0.000345677
74	212741_at	MAOA	3.891191741	0.000101467
75	205466_s_at	HS3ST1	3.854215088	9.35498E-06
76	207165_at	HMMR	3.840534839	0.000101055
77	206026_s_at	TNFAIP6	3.797444912	0.002618793
78	211362_s_at	SERPINB13	3.737353563	0.000705937
79	210314_x_at	TNFSF13	3.690262683	9.86407E-05
80	219100_at	OBFC1	3.633396063	0.000456639
81	206354_at	SLCO1B3	3.620075522	0.000117206
82	203560_at	GGH	3.597109147	8.25373E-05
83	218662_s_at	NCAPG	3.592034918	0.000179523
84	204491_at	PDE4D	3.545247609	0.000249339
85	214452_at	BCAT1	3.542895645	8.02782E-05
86	204464_s_at	EDNRA	3.532521126	8.25026E-05
87	219795_at	SLC6A14	3.517038598	0.000307605
88	217523_at	CD44	3.488896755	0.002563885
89	209500_x_at	TNFSF12-TNFS	3.483868669	3.32386E-05
90	204162_at	NDC80	3.445287461	9.43543E-05
91	201884_at	CEACAM5	3.443119917	0.000306676
92	201925_s_at	CD55	3.423048695	0.000161639
93	219090_at	SLC24A3	3.409592176	0.000332685
94	209709_s_at	HMMR	3.397067667	0.00141203
95	205659_at	HDAC9	3.36353568	0.000553566
96	205880_at	PRKD1	3.345251952	0.000975727
97	209773_s_at	RRM2	3.332007021	0.003544541
98	213441_x_at	SPDEF	3.330009744	0.0003584
99	218663_at	NCAPG	3.280511228	0.001232652
100	201926_s_at	CD55	3.275188012	0.000119627
101	202237_at	NNMT	3.259079004	0.000439007

102	209714_s_at	CDKN3	3.232033628	0.001787012
103	207526_s_at	IL1RL1	3.222962546	0.034056712
104	206424_at	CYP26A1	3.194703866	0.000191264
105	207317_s_at	CASQ2	3.157791382	0.034615872
106	211361_s_at	SERPINB13	3.131280524	0.000111471
107	205190_at	PLS1	3.108144199	4.78949E-05
108	217272_s_at	SERPINB13	3.102370968	0.000630598
109	218943_s_at	DDX58	3.091812947	0.000664229
110	204404_at	SLC12A2	3.075395984	0.000182031
111	219531_at	CEP72	3.054832749	0.000334762
112	218039_at	NUSAP1	3.027806719	0.001451674
113	201858_s_at	SRGN	3.026035135	0.004452472
114	205818_at	DBC1	3.024077128	0.002677641
115	219493_at	SHCBP1	3.01595254	0.000113556
116	205141_at	ANG	2.987128648	0.006085333
117	212909_at	LYPD1	2.980205985	0.000456999
118	206835_at	STATH	2.953405761	0.000352066
119	218802_at	CCDC109B	2.949605369	0.002910319
120	206488_s_at	CD36	2.928023829	0.033652844
121	218963_s_at	KRT23	2.897217094	0.000424556
122	210118_s_at	IL1A	2.896291739	0.000741291
123	202411_at	IFI27	2.887611973	0.001763299
124	204341_at	TRIM16	2.874641907	0.002480041
125	204962_s_at	CENPA	2.87251424	0.001395559
126	202207_at	ARL4C	2.868463577	0.000448051
127	204463_s_at	EDNRA	2.859579496	7.27223E-05
128	219836_at	ZBED2	2.856760693	0.00120168
129	203418_at	CCNA2	2.85259048	0.000162488
130	221218_s_at	TPK1	2.830177871	0.000501817
131	205158_at	RNASE4	2.823812237	0.0241454
132	201291_s_at	TOP2A	2.823729032	0.000516671
133	209499_x_at	TNFSF12-TNFS	2.81293983	0.000567922
134	203764_at	DLGAP5	2.808016015	0.000338862
135	220133_at	ODAM	2.80763574	0.011218703
136	218532_s_at	FAM134B	2.793717234	0.000270046
137	221841_s_at	KLF4	2.755483039	0.002265998
138	211303_x_at	FOLH1B	2.752907581	0.007339388
139	220779_at	PADI3	2.748943079	0.001800218
140	205185_at	SPINK5	2.747460774	0.000542493
141	210601_at	CDH6	2.740446132	0.007503274
142	203240_at	FCGBP	2.721616383	0.107840555
143	202037_s_at	SFRP1	2.716781102	0.000116859
144	221060_s_at	TLR4	2.704581725	0.001851121
145	205533_s_at	CDH6	2.695233654	0.004538295
146	210602_s_at	CDH6	2.692392386	0.004720587
147	219734_at	SIDT1	2.691571745	0.002245499
148	213107_at	TNIK	2.69135825	0.000638738
149	218510_x_at	FAM134B	2.678628265	0.000306742
150	206025_s_at	TNFAIP6	2.652907265	0.006314985
151	213397_x_at	RNASE4	2.63224088	0.013184395
152	212014_x_at	CD44	2.625700981	0.000968009
153	210916_s_at	CD44	2.601985888	0.000564212
154	219287_at	KCNMB4	2.594187596	0.001525576

155	207820_at	ADH1A	2.592120255	0.000199664
156	202503_s_at	KIAA0101	2.591139031	0.000574762
157	202238_s_at	NNMT	2.588524326	0.001169516
158	205381_at	LRRC17	2.581753788	0.001340533
159	214163_at	HSPB11	2.567556616	0.029957392
160	217080_s_at	HOMER2	2.567344535	0.005444986
161	203824_at	TSPAN8	2.558105493	0.030993483
162	202206_at	ARL4C	2.556040877	0.000970143
163	220658_s_at	ARNTL2	2.553528179	0.000308357
164	213226_at	CCNA2	2.542020964	0.000247408
165	218935_at	EHD3	2.538505481	0.000498233
166	205034_at	CCNE2	2.536683578	0.000224816
167	207414_s_at	PCSK6	2.534559325	0.000568689
168	209835_x_at	CD44	2.506575781	0.00069114
169	202201_at	BLVRB	2.500143173	0.00026149
170	211828_s_at	TNIK	2.497424474	0.001170302
171	204929_s_at	VAMP5	2.492768399	0.000430933
172	205042_at	GNE	2.475239253	0.000261364
173	211495_x_at	TNFSF12-TNFS	2.474171624	0.000355847
174	210413_x_at	SERPINB3	2.435829625	0.000273813
175	204026_s_at	ZWINT	2.433483627	0.000623148
176	220266_s_at	KLF4	2.43140635	0.013568556
177	201292_at	TOP2A	2.424224042	0.001340451
178	221011_s_at	LBH	2.417377226	0.000465379
179	206421_s_at	SERPINB7	2.416599889	0.000271611
180	218009_s_at	PRC1	2.402493219	0.00097354
181	205532_s_at	CDH6	2.401100648	0.003023213
182	205214_at	STK17B	2.400382583	0.001832396
183	204126_s_at	CDC45	2.397528218	0.000913467
184	202516_s_at	DLG1	2.391710273	0.001414948
185	217208_s_at	DLG1	2.373457025	0.004203765
186	213880_at	LGR5	2.37318103	0.007675212
187	205084_at	BCAP29	2.368857099	0.002243499
188	201890_at	RRM2	2.366426976	0.001208245
189	204490_s_at	CD44	2.365519519	0.000968319
190	220260_at	TBC1D19	2.363627511	0.000822027
191	205968_at	KCNS3	2.36048555	0.006819801
192	205046_at	CENPE	2.360382834	0.002663065
193	209904_at	TNNC1	2.336777103	0.047309768
194	217882_at	TMEM111	2.332329276	0.000689428
195	201666_at	TIMP1	2.320919991	0.175074594
196	207826_s_at	ID3	2.305169825	0.000498304
197	204489_s_at	CD44	2.30115906	0.001369973
198	208510_s_at	PPARG	2.299999181	0.001339388
199	218684_at	LRRC8D	2.299649215	0.000526454
200	205379_at	CBR3	2.290719215	0.002201924

Supplementary Table 4

72 hours treatment with IL-4

	id_chip	*entrez_gene*	*signed_ratio*	*FDR*
1	210809_s_at	POSTN	351.3891327	3.57205E-06
2	210521_s_at	FETUB	94.31071355	6.9655E-07
3	220622_at	LRRC31	91.76579253	4.52924E-08
4	214539_at	SERPINB10	46.98172307	4.41581E-07
5	206224_at	CST1	39.27397755	8.76725E-06
6	206529_x_at	SLC26A4	37.19121789	7.90259E-07
7	211478_s_at	DPP4	34.60171059	1.117E-06
8	209606_at	CYTIP	30.12625535	1.01213E-06
9	203717_at	DPP4	22.42640843	1.77889E-06
10	203240_at	FCGBP	21.53614175	0.000320572
11	209301_at	CA2	21.07256318	1.75812E-06
12	207328_at	ALOX15	18.5685403	2.16376E-06
13	203716_s_at	DPP4	18.44863101	4.53041E-07
14	205860_x_at	FOLH1	17.39930442	8.17601E-07
15	206932_at	CH25H	17.32952717	1.3657E-05
16	201884_at	CEACAM5	16.15705301	8.81109E-07
17	203913_s_at	HPGD	15.03897956	8.45779E-06
18	211548_s_at	HPGD	14.80206425	1.78125E-06
19	211549_s_at	HPGD	14.53157502	1.02763E-05
20	203434_s_at	MME	13.99761782	1.54593E-07
21	207367_at	ATP12A	13.88696204	8.50257E-07
22	218804_at	ANO1	13.71188962	2.16117E-06
23	203914_x_at	HPGD	13.58975492	2.04309E-06
24	204121_at	GADD45G	13.12812443	2.2272E-06
25	215363_x_at	FOLH1	12.99624478	8.5529E-07
26	206432_at	HAS2	12.62183979	0.003996647
27	206942_s_at	PMCH	12.46647036	1.30271E-06
28	219564_at	KCNJ16	11.96877975	0.000363872
29	214981_at	POSTN	11.7927711	1.00529E-05
30	219403_s_at	HPSE	11.65083428	3.70169E-07
31	206343_s_at	NRG1	11.04821878	5.81761E-07
32	203824_at	TSPAN8	10.7926978	7.53724E-07
33	213975_s_at	LYZ	10.44245395	2.30747E-06
34	206994_at	CST4	9.989388754	0.002365357
35	210107_at	CLCA1	9.815702359	0.00189741
36	206262_at	ADH1C	9.271420187	2.25968E-05
37	57588_at	SLC24A3	9.211339233	0.000157462
38	208605_s_at	NTRK1	9.073920928	1.52792E-05
39	206382_s_at	BDNF	8.756392413	7.75615E-05
40	220192_x_at	SPDEF	8.635160618	2.97649E-07
41	209555_s_at	CD36	8.464698921	2.7291E-06
42	210001_s_at	SOCS1	8.377097779	3.79625E-07
43	216881_x_at	PRB4	7.861107084	0.019937746
44	208126_s_at	CYP2C18	7.833872194	7.37794E-07
45	205141_at	ANG	7.807502489	5.16032E-05
46	219090_at	SLC24A3	7.736553089	0.000162282
47	206424_at	CYP26A1	7.614507834	3.93162E-05
48	205158_at	RNASE4	7.384666776	2.19476E-05

49	209267_s_at	SLC39A8	7.327859363	1.68174E-05
50	212942_s_at	KIAA1199	7.224226353	9.69222E-07
51	206488_s_at	CD36	7.193859885	2.70602E-06
52	218532_s_at	FAM134B	7.149862557	2.02322E-05
53	203435_s_at	MME	7.120409507	7.6054E-06
54	219727_at	DUOX2	6.943438434	8.60824E-06
55	204614_at	SERPINB2	6.896825399	4.01659E-05
56	214404_x_at	SPDEF	6.858826691	5.91768E-07
57	206172_at	IL13RA2	6.789643566	0.000156779
58	219764_at	FZD10	6.789150128	3.96125E-05
59	215103_at	CYP2C18	6.584360308	1.0196E-06
60	218510_x_at	FAM134B	6.560819762	5.8757E-05
61	213397_x_at	RNASE4	6.273718021	1.68014E-05
62	210597_x_at	PRB1	6.238394675	0.023316414
63	211531_x_at	PRB1	6.123726679	0.010567365
64	221874_at	KIAA1324	6.112749725	0.000466658
65	207752_x_at	PRB1	6.094747115	0.005952485
66	219508_at	GCNT3	6.092953921	2.85513E-05
67	205969_at	AADAC	6.046113909	6.0322E-05
68	213974_at	ADAMTSL3	5.852083611	5.06818E-06
69	219869_s_at	SLC39A8	5.755516034	1.13687E-05
70	214907_at	CEACAM21	5.731220001	2.31384E-05
71	201859_at	SRGN	5.717929479	0.000120887
72	220779_at	PADI3	5.691398865	9.84119E-05
73	220017_x_at	CYP2C9	5.680880712	8.58696E-06
74	221223_x_at	CISH	5.648092465	1.23043E-05
75	204515_at	HSD3B1	5.605150338	0.040849595
76	219100_at	OBFC1	5.556214092	1.40507E-05
77	201926_s_at	CD55	5.533423879	1.02841E-06
78	211303_x_at	FOLH1B	5.513316816	2.24272E-06
79	204491_at	PDE4D	5.258260696	1.6845E-05
80	204389_at	MAOA	5.218443724	1.96948E-05
81	204388_s_at	MAOA	5.167705513	1.40403E-05
82	213441_x_at	SPDEF	5.116605119	2.20398E-06
83	214452_at	BCAT1	5.086859391	2.30605E-06
84	218976_at	DNAJC12	5.050663555	1.78691E-06
85	212741_at	MAOA	4.984875697	1.05251E-05
86	204404_at	SLC12A2	4.964436839	9.22079E-07
87	212158_at	SDC2	4.955752709	0.002758362
88	208555_x_at	CST2	4.939117413	0.001727787
89	207958_at	UGT2A1	4.905029873	0.000331892
90	205466_s_at	HS3ST1	4.875300639	7.96367E-07
91	201858_s_at	SRGN	4.642902993	5.11378E-05
92	219334_s_at	OBFC2A	4.592044172	4.15231E-06
93	210619_s_at	HYAL1	4.590372185	1.51788E-06
94	201925_s_at	CD55	4.562644061	3.52973E-06
95	214068_at	BEAN1	4.410026003	0.005944295
96	207820_at	ADH1A	4.371393014	0.001503961
97	209340_at	UAP1	4.370822144	1.77291E-06
98	205676_at	CYP27B1	4.332938124	0.010956869
99	206037_at	CCBL1	4.311052925	3.24292E-05
100	211906_s_at	SERPINB4	4.309092077	3.62245E-05
101	220266_s_at	KLF4	4.306017144	0.000318274

102	218943_s_at	DDX58	4.238024889	1.35647E-06
103	204396_s_at	GRK5	4.194952011	0.004955955
104	209904_at	TNNC1	4.145427404	0.000454207
105	221796_at	NTRK2	4.135128187	0.016154766
106	210314_x_at	TNFSF13	4.05473552	1.52751E-06
107	219429_at	FA2H	4.05111113	8.25447E-06
108	212233_at	MAP1B	4.041921538	0.001714335
109	203560_at	GGH	3.990261559	2.17093E-06
110	214385_s_at	MUC5AC	3.98167134	0.001756232
111	216504_s_at	SLC39A8	3.978583691	3.6533E-05
112	201666_at	TIMP1	3.971231838	0.002886724
113	209500_x_at	TNFSF12-TNFS	3.963657574	2.18785E-06
114	220446_s_at	CHST4	3.950758287	2.19197E-06
115	206766_at	ITGA10	3.836388432	0.000957803
116	212154_at	SDC2	3.805239531	0.004556647
117	205818_at	DBC1	3.777167016	0.000718308
118	210029_at	IDO1	3.77101294	4.24779E-05
119	203895_at	PLCB4	3.766591463	5.63044E-06
120	219619_at	DIRAS2	3.720428854	0.003789364
121	205185_at	SPINK5	3.710453561	5.9724E-05
122	219728_at	MYOT	3.676224075	1.06466E-05
123	203896_s_at	PLCB4	3.672249494	8.80722E-06
124	221841_s_at	KLF4	3.666025426	4.78142E-05
125	221241_s_at	BCL2L14	3.647868204	9.14215E-05
126	219795_at	SLC6A14	3.643543409	2.21321E-06
127	217523_at	CD44	3.625152087	1.90034E-05
128	205267_at	POU2AF1	3.624687085	6.0636E-05
129	217123_x_at	PMCHL1	3.601612596	6.54882E-05
130	205042_at	GNE	3.555414847	7.75965E-05
131	209417_s_at	IFI35	3.552454349	8.56038E-06
132	46323_at	CANT1	3.529956633	3.76658E-05
133	212295_s_at	SLC7A1	3.512572329	0.026622582
134	200628_s_at	WARS	3.504542639	0.000174307
135	213695_at	PON3	3.498873949	0.000383815
136	214303_x_at	MUC5AC	3.491261736	0.002345539
137	221218_s_at	TPK1	3.471076099	3.76185E-05
138	218802_at	CCDC109B	3.466946243	0.000104642
139	200629_at	WARS	3.456074975	4.31461E-05
140	204284_at	PPP1R3C	3.429867107	0.000195919
141	220133_at	ODAM	3.40431198	0.002517014
142	206421_s_at	SERPINB7	3.401417851	0.000169782
143	203971_at	SLC31A1	3.38525767	6.76919E-05
144	204241_at	ACOX3	3.383060487	0.000565104
145	204464_s_at	EDNRA	3.373602868	4.14179E-06
146	214971_s_at	ST6GAL1	3.34807012	1.59736E-05
147	219142_at	RASL11B	3.326146971	0.000521371
148	221060_s_at	TLR4	3.314593132	4.08855E-05
149	205190_at	PLS1	3.312290812	3.12009E-06
150	216605_s_at	CEACAM21	3.248837319	0.000114668
151	211689_s_at	TMPRSS2	3.248033372	0.000138174
152	207414_s_at	PCSK6	3.217397699	1.46964E-05
153	219918_s_at	ASPM	3.208208711	0.000233549
154	216258_s_at	SERPINB13	3.19900445	0.000173515

155	209696_at	FBP1	3.187191932	0.000383861
156	221732_at	CANT1	3.186916939	3.7205E-05
157	204242_s_at	ACOX3	3.177120988	0.002024077
158	209521_s_at	AMOT	3.166737763	1.37569E-05
159	210602_s_at	CDH6	3.165865888	0.001223648
160	204687_at	PARM1	3.159257358	0.000943724
161	205552_s_at	OAS1	3.148928584	2.23536E-05
162	205876_at	LIFR	3.143778652	9.44122E-06
163	219597_s_at	DUOX1	3.143217721	1.06307E-05
164	204972_at	OAS2	3.136909191	0.000809807
165	218963_s_at	KRT23	3.135139959	0.000230408
166	209499_x_at	TNFSF12-TNFS	3.120732912	3.71677E-05
167	219148_at	PBK	3.119575595	0.00019561
168	204623_at	TFF3	3.101011487	1.60949E-05
169	209930_s_at	NFE2	3.096393827	9.3514E-05
170	210064_s_at	UPK1B	3.08774194	3.59413E-06
171	204463_s_at	EDNRA	3.081452315	0.000196594
172	210037_s_at	NOS2	3.077074407	0.000119225
173	212290_at	SLC7A1	3.066026101	0.018015102
174	205659_at	HDAC9	3.06369845	8.52102E-05
175	211361_s_at	SERPINB13	3.062127906	7.25857E-06
176	204971_at	CSTA	3.054117051	0.000107003
177	206354_at	SLCO1B3	3.045942636	0.000167644
178	221011_s_at	LBH	3.04447676	4.32016E-05
179	205630_at	CRH	3.043981733	0.001081164
180	211495_x_at	TNFSF12-TNFS	3.03793202	9.48575E-05
181	206835_at	STATH	3.032667154	0.00210808
182	202869_at	OAS1	3.023344824	2.6872E-05
183	213663_s_at	---	3.023008859	0.000397604
184	202112_at	VWF	3.001548846	0.001200625
185	200973_s_at	TSPAN3	3.000412946	1.6525E-05
186	205590_at	RASGRP1	2.960552116	0.002417792
187	202411_at	IFI27	2.960098499	2.32264E-05
188	214040_s_at	GSN	2.959697348	0.000474195
189	210601_at	CDH6	2.937080256	0.006407294
190	202079_s_at	TRAK1	2.913851911	0.00075954
191	204929_s_at	VAMP5	2.90647273	4.22532E-05
192	205533_s_at	CDH6	2.89784493	0.001511462
193	211362_s_at	SERPINB13	2.870052258	0.000131383
194	205880_at	PRKD1	2.869068301	0.001474963
195	204753_s_at	HLF	2.865460996	0.005925664
196	202201_at	BLVRB	2.864684457	1.52181E-05
197	204017_at	KDELR3	2.859783603	0.000484943
198	204698_at	ISG20	2.847005331	4.35995E-05
199	204161_s_at	ENPP4	2.821796692	3.96898E-05
200	204160_s_at	ENPP4	2.819094963	1.1569E-05
